# Engineered pattern recognition receptors enhance broad-spectrum plant resistance

**DOI:** 10.1038/s41587-025-02858-8

**Published:** 2025-10-13

**Authors:** Yuankun Yang, Christina E. Steidele, Xianlong Huang, Birgit Löffelhardt, Lili Huang, Jonathan D. G. Jones, Thorsten Nürnberger, Pingtao Ding, Andrea A. Gust

**Affiliations:** 1 Department of Plant Biochemistry, Center of Plant Molecular Biology (ZMBP), Eberhard-Karls-University of Tübingen, Tübingen, Germany; 2 Institute of Biology Leiden, Leiden University, Leiden, The Netherlands; 3 Chair of Phytopathology, TUM School of Life Sciences, Technische Universität München, Freising-Weihenstephan, Germany; 4 State Key Laboratory of Crop Stress Biology for Arid Areas, College of Plant Protection, Northwest A&F University, Yangling, China; 5 The Sainsbury Laboratory, University of East Anglia, Norwich, UK

**Keywords:** Pattern recognition receptors in plants, Molecular engineering in plants

## Abstract

Conventional plant resistance breeding has primarily focused on intracellular immune receptors, while cell-surface pattern recognition receptors (PRRs) have been underexplored because of their comparatively modest contributions to resistance. However, PRRs offer untapped potential for crop improvement. Here we demonstrate that the *Arabidopsis* receptor-like protein RLP23, which recognizes molecular patterns from three distinct microbial kingdoms, confers broad-spectrum resistance when introduced into the Solanaceae crop tomato. We also identify the C-terminal domain of RLPs as crucial for ensuring compatibility and efficacy during heterologous expression. Targeted engineering of the C-terminal domain significantly enhances RLP23 and its use, enabling transfer of robust resistance against bacterial, fungal and oomycete pathogens to tomato plants without compromising yield. We extend this RLP engineering strategy to rice and poplar, highlighting its broad applicability. These findings establish a versatile framework for PRR-based engineering, opening additional avenues for sustainable crop protection.

## Abstract (web summary)

Engineering an immune receptor improves resistance to bacterial and fungal pathogens in a variety of plants.

## Main

Plants rely on a limited repertoire of immune receptors to combat diverse pathogens, classified into pattern recognition receptors (PRRs), which reside at the plasma membrane and initiate cell-surface immunity (pattern-triggered immunity), and intracellular nucleotide-binding leucine-rich repeat receptors (NLRs), which detect pathogen effectors to activate effector-triggered immunity (ETI)^1^. Evolutionary divergence among plant species results in varying immune receptor distributions and resistance traits, making interspecies receptor transfer a promising strategy to boost crop resilience.

Over the past decades, many resistance genes, mainly from the NLR family, have been cloned and used in crop breeding. NLRs typically confer race-specific resistance by recognizing specific pathogen effectors but the rapid evolution of pathogens can render NLR-equipped plants vulnerable to newly emerging pathogen strains^2^. To address this, stacking multiple *NLR* genes has been explored as a strategy to enhance resistance^3^, although the continuous evolution of pathogens poses a challenge to the durability of such crops.

In contrast, PRRs recognize conserved pathogen-associated molecular patterns, which are crucial to microbial physiology and less prone to evolution, making them attractive for obtaining broad-spectrum resistance. Despite this advantage, PRRs have been underused in crop breeding because of their relatively modest immune responses compared with intracellular receptors. Only a few PRRs, such as the *Arabidopsis* receptor-like kinase (RLK) EF-TU RECEPTOR (EFR), have been used to enhance bacterial disease when expressed in crops like wheat, apple and tomato^4,5^.

Leucine-rich repeat receptor-like proteins (LRR-RLPs) also show potential for improving disease resistance. For example, transferring the elicitin receptor ELICITIN RESPONSE from wild potato to cultivated varieties confers resistance to *Phytophthora* *infestans*^6^. Similarly, introducing the xyloglucanase receptor RESPONSE TO XEG1 from *Nicotiana* *benthamiana* into wheat enhances resistance to *Fusarium* head blight^7^. However, these PRRs recognize specific pathogen classes only, limiting their broader application^6,7^.

Two *Arabidopsis* receptors, RLP23 and RLP30, detect molecular patterns from all three microbial kingdoms^8,9^. RLP23 senses necrosis-inducing and ethylene-inducing peptide 1-like proteins (NLPs) from fungi, oomycetes and specific bacterial species, while RLP30 recognizes small cysteine-rich proteins (SCPs) from fungal and oomycete pathogens, along with an unidentified *Pseudomonas* pattern. This broad recognition makes RLP23 and RLP30 promising candidates for PRR-based crop engineering. Notably, transgenic potato expressing RLP23 shows elevated resistance to the fungal pathogen *Sclerotinia* *sclerotiorum* and the oomycete *P*. *infestans*^8^.

However, heterologous expression of RLPs often faces compatibility issues, requiring coexpression of additional receptor complex components for optimal functionality. For instance, cotransformation of RLP30 with the adaptor kinase SUPPRESSOR OF BIR1 1 (SOBIR1) from *Arabidopsis* *thaliana* in *Nicotiana* *tabacum* achieved stronger resistance compared with RLP30 alone^9^. Yet, overexpression of coreceptors can result in autoimmune phenotypes, such as dwarfism and premature flowering^10^. While recent advances in NLR engineering have expanded their recognition spectrum, as demonstrated for the rice NLR Pik-1 (ref. ^11^), they remain pathogen specific. In contrast, engineering PRRs for broad-spectrum recognition while enhancing their functionality is an underexplored approach for crop breeding. Here, we show that the C-terminal domains (CT domain) of RLPs determine compatibility during heterologous expression. By modifying the C terminus of RLP23, we enhance tomato resistance to bacteria, fungi and oomycetes, without compromising yield. Notably, this PRR modification strategy is also applicable to the monocot species rice and the perennial poplar. Our findings provide a scalable framework for engineering PRRs to achieve broad-spectrum resistance, offering a sustainable approach for crop improvement.

## Results

### RLP23 transfer enhances tomato resistance to multiple pathogens

The *Arabidopsis* receptor RLP23 recognizes the conserved immunogenic epitope nlp20, which is present in pathogenic fungi, oomycetes and certain bacteria^12^, making it a promising tool for crop improvement. To explore this potential, we generated a transgenic tomato line stably expressing RLP23 fused with GFP under the constitutive 35S promoter (*p35S::RLP23–GFP*). Western blot analyses confirmed successful expression of RLP23 (Supplementary Fig. 1a,b).

**Fig. 1:**
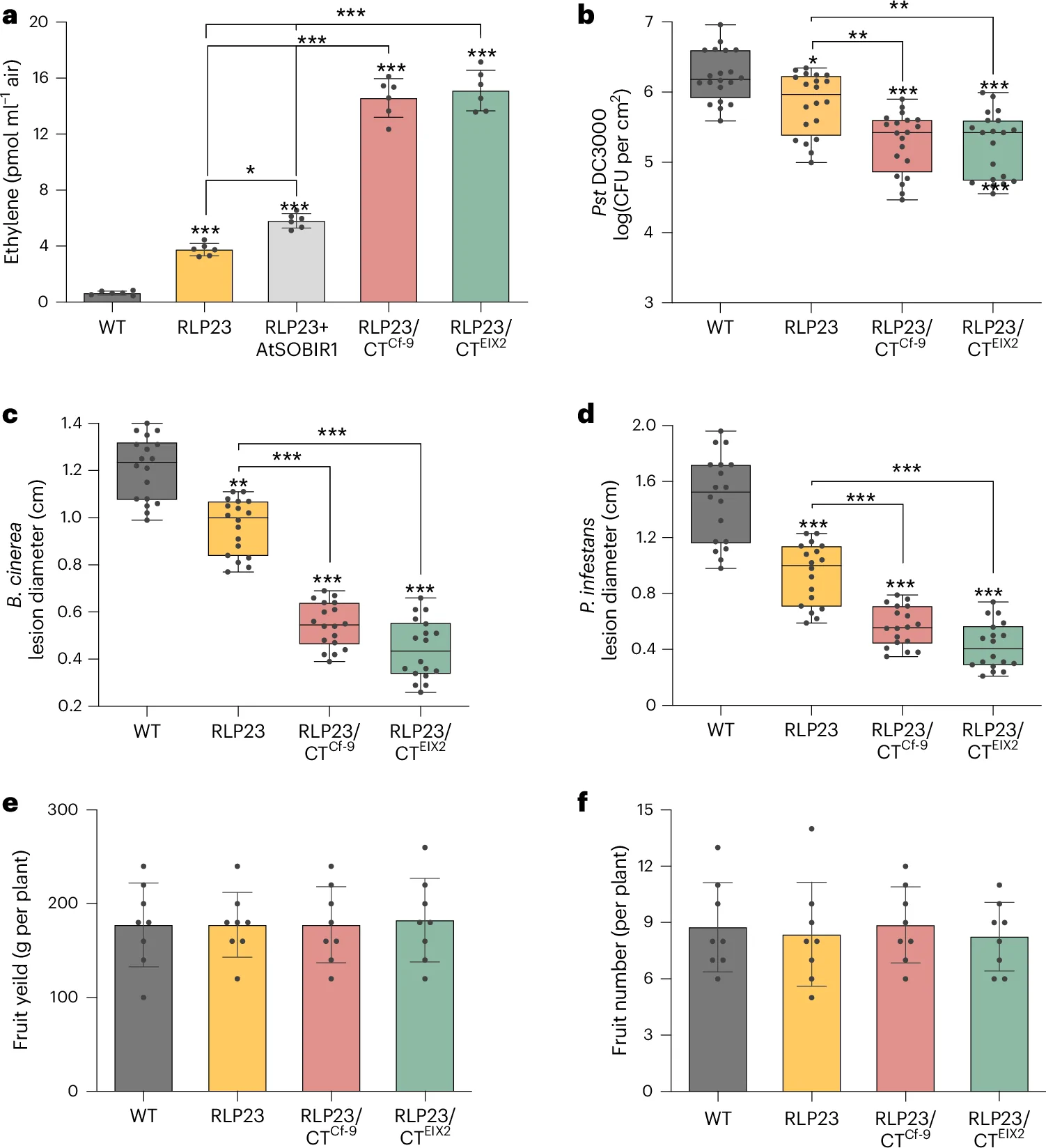
Expression of RLP23 chimeras in tomato confers increased defense without compromising yield. **a**, Ethylene accumulation in WT and transgenic tomato plants expressing RLP23, RLP23 with AtSOBIR1, RLP23/CT^Cf-9^ or RLP23/CT^EIX2^, measured after 4 h of treatment with 2 µM nlp20. **b**, Bacterial growth of *Pst* DC3000 in WT and transgenic tomato plants, quantified as CFU in leaf extracts 3 days after inoculation. **c**,**d**, Lesion diameters on WT and transgenic tomato leaves infected with *B*. *cinerea* (**c**) or *P*. *infestans* (**d**), assessed 2 days after drop inoculation. **e**,**f**, Total weight (**e**) and number (**f**) of mature fruits collected per tomato plant. Data in **a**, **e** and **f** are presented as the mean ± s.d. (*n* = 6 for **a**; *n* = 8 for **e** and **f**). For **b**–**d**, data are displayed as box plots (center line, median; bounds of box, the first and third quartiles; whiskers, 1.5 times the interquartile range; error bar, minima and maxima; *n* = 20 for **b**; *n* = 18 for **c** and **d**). Data were analyzed using a two-way analysis of variance (ANOVA) with Tukey’s test. Statistically significant differences from WT are indicated (*P ≤ 0.05, **P ≤ 0.01 and ***P ≤ 0.001). Each experiment was repeated three times with consistent results. Source data

Compared with wild-type (WT) tomato plants, the transgenic line showed increased ethylene production in response to nlp20 stimulation (Fig. 1a). To assess whether RLP23-mediated recognition of nlp20 enhances immunity, we challenged the transgenic plants with the bacterial pathogen *Pseudomonas* *syringae* pv. *tomato* (*Pst*) DC3000, the fungal pathogen *Botrytis* *cinerea* and the oomycete *P*. *infestans*. Notably, transgenic tomatoes displayed enhanced antibacterial immunity against *Pst* DC3000, despite the apparent absence of NLPs in this pathogen (Fig. 1b). We hypothesize that RLP23 may detect unknown bacterial ligands, similar to how RLP30 recognizes conserved cysteine-rich proteins from both fungi and oomycetes, as well as structurally unrelated bacterial ligands^9^. Additionally, RLP23-expressing plants showed significantly smaller disease lesions upon infection with both *B*. *cinerea* and *P*. *infestans* compared with WT plants (Fig. 1c,d), confirming that RLP23 expression confers broad-spectrum resistance to diverse pathogens in tomato.

To further explore whether coexpression of RLP23 with the adaptor protein AtSOBIR1 could boost immunity, we generated a transgenic tomato line expressing both RLP23 and AtSOBIR1. Coexpression resulted in a modest increase in nlp20-induced ethylene production compared with RLP23 expression alone (Fig. 1a). However, AtSOBIR1 expression also caused dwarfism, likely because of autoimmune activation (Supplementary Fig. 1a). This phenotype contrasts with *N*. *tabacum*, where coexpression of RLP30 and AtSOBIR1 did not affect plant growth^9^, suggesting species-specific differences in AtSOBIR1-mediated immune regulation. While coexpression with AtSOBIR1 slightly enhanced immune signaling, its negative impact on growth limits its practical application for crop improvement. Overall, these results demonstrate that stable ectopic expression of RLP23 in tomato promotes broad-spectrum resistance to destructive bacterial, fungal and oomycete pathogens.

### Full RLP functionality in heterologous plants depends on the CT domains

RLPs contain a short C-terminal intracellular domain (IC domain) (Supplementary Fig. 2) but its role in immune signaling remains unclear. To investigate its contribution to RLP-mediated immunity, we introduced a truncated RLP23 variant lacking the IC domain (RLP23ΔIC) into the *Arabidopsis* *rlp23*-1 mutant. However, deletion of the IC domain did not impair ethylene accumulation in response to nlp20 treatment (Supplementary Fig. 3a,b). Surprisingly, transient expression of RLP23ΔIC in *N*. *benthamiana* resulted in a significantly weaker ethylene response to nlp20 compared with full-length RLP23 (Supplementary Fig. 3c,g), suggesting that the IC domain has a species-dependent role in immune activation.

We also examined RLP30, an SCP receptor with a short IC domain of 23 aa^9^. In an *N*. *benthamiana* line lacking the SCP receptor RE02 (ΔRE02), expression of full-length RLP30 reconstituted SCP-triggered ethylene production, whereas the truncated variant lacking the intracellular structure (RLP30ΔIC) failed to restore the responses to RLP30 WT levels (Supplementary Fig. 3d,h).

Interestingly, the IC domains of RLPs harbor putative phosphorylation sites—four in RLP30 (T761, T783, T784 and S785) and two in RLP23 (S872 and Y873) (Supplementary Fig. 2b). To assess their functional relevance, we generated phospho-dead mutants by substituting these residues with alanine or phenylalanine (T761A;T783A;T784A;S785A in RLP30^4mut^ and S872A;Y873F in RLP23^2mut^). Immune responses triggered by both SCP and nlp20 remained unchanged in *N*. *benthamiana* plants expressing the mutant receptors (Supplementary Fig. 3e,f,i,j), indicating that RLP-mediated immunity operates independently of IC domain phosphorylation.

In addition to the IC domain, the CT region of RLPs contains transmembrane (TM) and juxtamembrane (JM) domain (Supplementary Fig. 2). To assess their contribution to immune signaling, we generated two RLP23 variants: one lacking both TM and IC and another with a complete CT deletion (CT, IC + TM + JM) (Supplementary Fig. 4a). Ethylene accumulation induced by nlp20 was comparable in *N*. *benthamiana* expressing RLP23ΔIC + TM and RLP23ΔIC, whereas additional deletion of the JM domain further compromised immune responses (Supplementary Fig. 4b). Similar results were obtained with RLP30 CT truncations (Supplementary Fig. 4c). These findings highlight the essential role of the CT domains in RLP-mediated immunity in heterologous plant systems.

### CT swaps modulate RLP functionality

The reduced activity upon CT deletions of RLP23 and RLP30 in *N*. *benthamiana* underscores the critical role of the C terminus in heterologous expression systems. To assess whether CT domains from solanaceous RLPs could enhance RLP23 functionality in the Solanaceae family, we selected two well-characterized tomato RLPs, EIX2 and Cf-9, as CT donors because of their overall structural similarity to RLP23 (Supplementary Fig. 2). EIX2 detects ethylene-inducing xylanase, while Cf-9 recognizes the *Cladosporium* *fulvum* effector protein Avr9 (refs. ^13,14^).

**Fig. 2:**
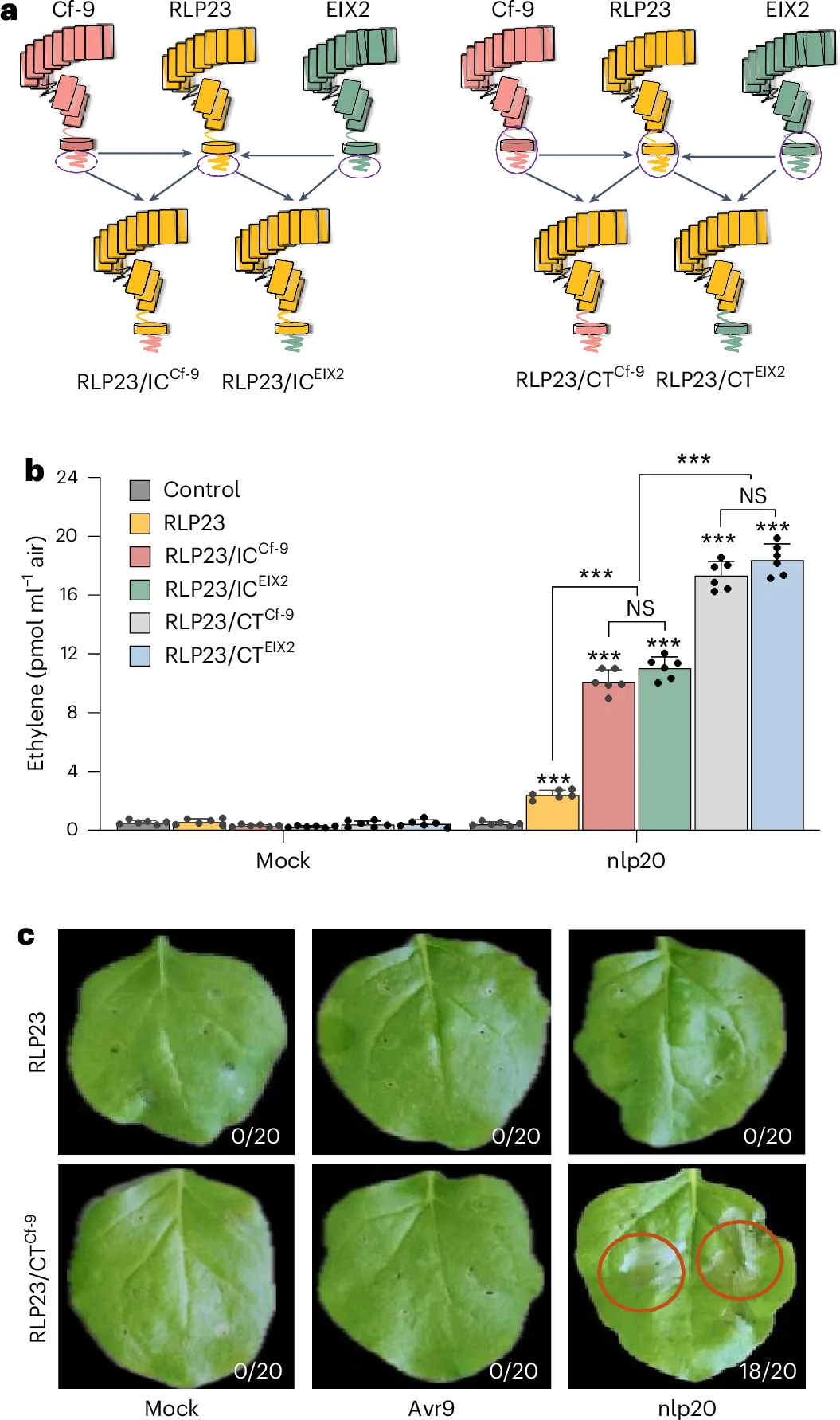
CT domain swaps result in RLP23 activity differences in *N*. *benthamiana.* **a**, Schematic representation of RLP23, Cf-9 and EIX2, along with their chimeric variants featuring reciprocal IC or CT domain swaps. **b**, Ethylene accumulation in *N*. *benthamiana* leaves transiently expressing GFP (control), RLP23–GFP, RLP23/IC^Cf-9^–GFP, RLP23/IC^EIX2^–GFP, RLP23/CT^Cf-9^–GFP or RLP23/CT^EIX2^–GFP, measured 4 h after treatment with water (mock) or 2 µM nlp20. Data are presented as the mean ± s.d. (*n* = 6) and were analyzed using a two-way ANOVA with Tukey’s test. Statistically significant differences from control plants are indicated (***P ≤ 0.001; NS, not significant). **c**, Cell death development in *N*. *benthamiana* leaves transiently expressing RLP23–GFP or RLP23/CT^Cf-9^–GFP, 72 h after infiltration with water (mock), 20 µM nlp20 or 100 µl of apoplastic fluid with Avr9. The number of necrotic leaves is presented relative to the total number of infiltrated leaves. Each experiment was repeated three times with similar results. Source data

We constructed chimeric receptors by swapping the IC or CT domains of RLP23, EIX2 and Cf-9 (Fig. 2a and Supplementary Figs. 5 and 6). Expression of RLP23/IC^EIX2^ and RLP23/IC^Cf-9^ in *N*. *benthamiana* resulted in a fourfold increase in ethylene production in response to nlp20 treatment compared with WT RLP23 (Fig. 2b), indicating that the IC domains from solanaceous RLPs enhance RLP23-mediated immune responses.

Substitution of the entire RLP23 CT region with that of tomato RLPs further amplified its immune function in *N. benthamiana*, measurable by elevated ethylene production in response to nlp20 (Fig. 2b). A similar enhancement was observed upon editing the full CT region of RLP30, which led to stronger SCP-induced immune responses in ΔRE02 plants expressing RLP30/CT^EIX2^ or RLP30/CT^Cf-9^ (Supplementary Fig. 5b). In contrast, replacing the CT domains in EIX2 and Cf-9 with that of RLP23 significantly reduced their respective responses to EIX and Avr9 (Supplementary Fig. 6b,c), demonstrating that the CT domains contribute to receptor-specific immune signaling.

Interestingly, RLP23/CT^Cf-9^ expression induced nlp20-triggered cell death, a hallmark of ETI that was not previously observed in any plant following nlp20 treatment (Fig. 2c). Conversely, Cf-9/CT^RLP23^ did not produce the cell death response usually triggered by Avr9 (Supplementary Fig. 6e), indicating that the C terminus has a key role in regulating immune signaling outcomes. These findings highlight the functional importance of the C terminus in modulating RLP activity, enhancing immune responses and influencing cell death phenotypes in heterologous expression systems.

### Disease resistance in tomato is enhanced by RLP23 CT editing without yield loss

Building on the enhanced RLP23 functionality observed in *N*. *benthamiana*, we explored whether RLP23 chimeric receptors could improve disease resistance in tomato. The *RLP23/CT*^*EIX2*^ and *RLP23/CT*^*Cf-9*^ constructs were stably introduced into the tomato cultivar Moneymaker, which does not naturally respond to nlp20. Consistent with the results from *N*. *benthamiana*, transgenic tomato lines expressing the RLP23 hybrid receptors showed a substantial increase in nlp20-induced ethylene production and enhanced disease resistance (Fig. 1a–d). Compared with WT and RLP23-expressing plants, *RLP23/CT*^*EIX2*^ and *RLP23/CT*^*Cf-9*^ transgenic tomatoes exhibited significantly reduced bacterial growth after *Pst* DC3000 infection (Fig. 1b) and developed smaller disease lesions when challenged with the necrotrophic fungus *B*. *cinerea* (Fig. 1c) or the oomycete *P*. *infestans* (Fig. 1d).

The tradeoff between defense and growth has long been a key challenge in crop breeding^15^. In contrast to the dwarfism observed in plants coexpressing RLP23 with AtSOBIR1, tomato lines carrying *RLP23/CT*^*EIX2*^ and *RLP23/CT*^*Cf-9*^ showed no developmental defects, with growth patterns comparable to those of WT and RLP23-expressing plants (Supplementary Fig. 1a). Importantly, the enhanced resistance conferred by the engineered RLP23 receptors did not affect fruit yield. Both transgenic and WT tomato plants produced similar numbers and total weight of mature fruits (Fig. 1e,f). These findings demonstrate that RLP engineering can significantly enhance broad-spectrum resistance in tomato without compromising yield.

### Interaction of RLP23 with SOBIR1 is modulated by the CT domain

To understand how tomato RLP CT domains enhance RLP23 functionality in Solanaceae, we examined their impact on immune signaling. Consistent with the elevated nlp20-triggered ethylene levels observed in RLP23-expressing plants, we also detected significant upregulation of immune-related gene transcription upon nlp20 treatment. Genes such as *PR1a* and *RIPK* were strongly induced, with even higher expression levels in transgenic tomato lines expressing the chimeric RLP23/CT^EIX2^ (Supplementary Fig. 7a). Notably, these lines accumulated transcripts of genes typically associated with ETI, such as *EDS1*, *NRC2* and *NRC4a*, in response to nlp20 but not to treatment with flg22 (Supplementary Fig. 7a,b). These findings suggest that the immune response triggered by RLP23 variants in Solanaceae involves elements known to function in ETI pathways.

**Fig. 3:**
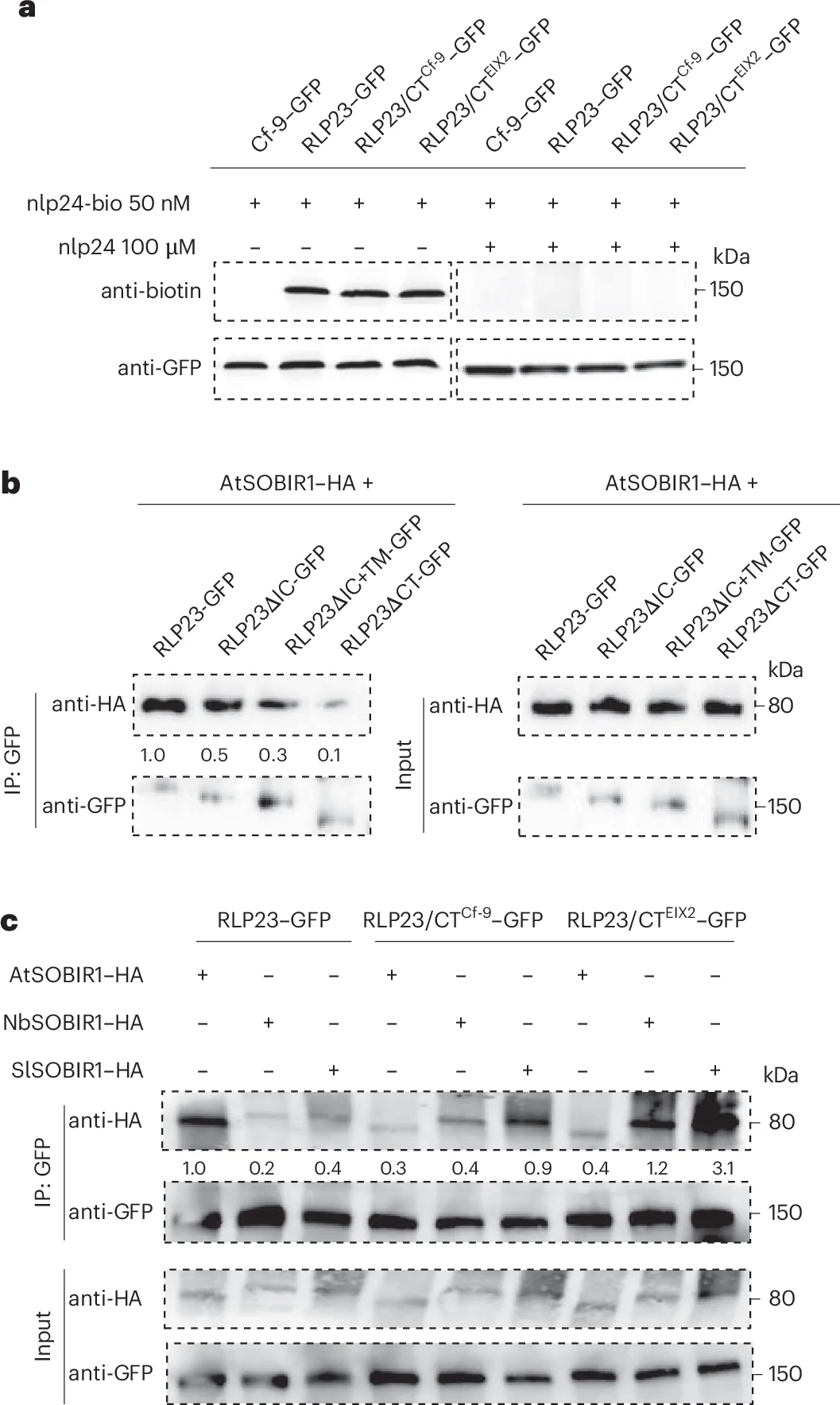
Replacement of RLP23 IC does not affect ligand binding but the interaction with SOBIR1. **a**, Ligand-binding assay in *N*. *benthamiana* leaves transiently expressing indicated GFP-tagged RLP variants. Leaves were treated with biotinylated nlp24 (nlp24-bio) in the presence (+) or absence (−) of unlabeled nlp24, followed by coimmunoprecipitation using a GFP-Trap. **b**, Coimmunoprecipitation assay in *N*. *benthamiana* leaves coexpressing GFP-tagged RLP23 variants with AtSOBIR1–HA. Leaf protein extracts (input) were subjected to coimmunoprecipitation with GFP beads (IP:GFP) and immunoblotting using indicated antibodies. **c**, Coimmunoprecipitation assay in *N*. *benthamiana* leaves coexpressing GFP-tagged RLP23 variants with AtSOBIR1–HA, NbSOBIR1–HA, or SlSOBIR1–HA. Leaf protein extracts (input) were subjected to coimmunoprecipitation with GFP beads (IP:GFP) and immunoblotting using tag-specific antibodies. Protein levels were quantified relative to AtSOBIR1–HA precipitated by RLP23–GFP, set as 1 (**b**,**c**). Representative results from three independent experiments are shown. Source data

To assess whether ligand-binding capacities were altered in the RLP23 chimeric proteins, we tested their interaction with biotinylated nlp24 (nlp24-bio), a functional analog of nlp20 that includes a C-terminal 4-aa extension. All tested variants, including WT RLP23, RLP23/CT^EIX2^ and RLP23/CT^Cf-9^, associated similarly with nlp24-bio. Excess of untagged nlp24 effectively displaced nlp24-bio from all RLP23 variants (Fig. 3a), indicating that CT domain replacement does not affect the ligand-binding properties of RLP23. Thus, the enhanced immune response is unlikely to be because of altered ligand recognition.

In addition, we evaluated the roles of RLP23 CT region in mediating interaction specificity with the adaptor kinase SOBIR1. Coimmunoprecipitation assays revealed that deletion of the IC or IC + TM domains markedly reduced RLP23–SOBIR1 interaction, while removal of the entire C terminus nearly abolished binding (Fig. 3b). Interestingly, chimeric receptors RLP23/CT^EIX2^ and RLP23/CT^Cf-9^ showed stronger association with SOBIR1 from *N*. *benthamiana* and tomato than with AtSOBIR1 (Fig. 3c and Supplementary Fig. 8). While WT RLP23 preferentially associated with AtSOBIR1, the chimeric variants exhibited the strongest association with SlSOBIR1 (Fig. 3c and Supplementary Fig. 8). These findings highlight the critical role of the C terminus in modulating RLP–SOBIR1 interactions and demonstrate that its targeted engineering can improve RLP23’s adaptation and integration into the immune signaling networks of heterologous plants.

### RLP23 expression in rice confers recognition of NLPs from blast fungi

**Fig. 4:**
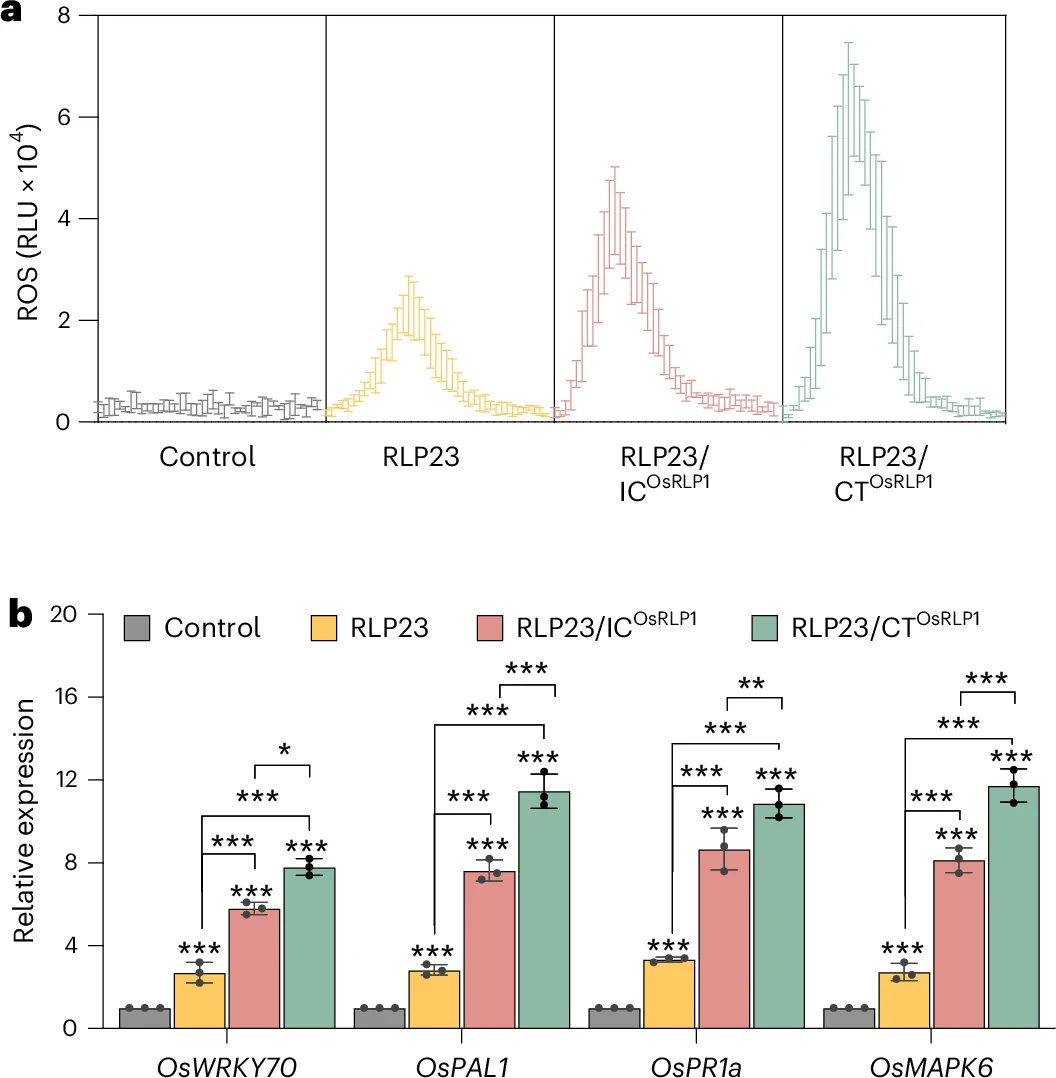
Expression of RLP23 chimeras enhances immune responses in rice. **a**, ROS production in rice protoplasts expressing GFP (control), RLP23–GFP, RLP23/IC^OsRLP1^–GFP or RLP23/CT^OsRLP1^–GFP, following treatment with 5 µM purified MoNLP1. Data are presented as relative light units (RLU) over 2 h. **b**, RT–qPCR analysis of rice protoplasts expressing GFP (control) or GFP-tagged RLP23 variants, treated with 2 µM MoNLP1 for 24 h. Expression levels of the indicated marker genes were quantified using gene-specific primers, normalized to *OsActin* transcript levels and presented relative to the control (set to 1). Data are presented as the mean ± s.d. (*n* = 3) and were analyzed using a two-way ANOVA with Tukey’s test. Statistically significant differences from the control are indicated (*P ≤ 0.05, **P ≤ 0.01 and ***P ≤ 0.001). Each experiment was performed three times with similar results. Source data

Given that *Arabidopsis* RLP23 conferred resistance in dicot plants such as tomato and tobacco (Figs. 1 and 2), we next investigated its potential to improve immunity in monocot crops. The rice blast fungus *Magnaporthe* *oryzae* encodes the NLP protein MoNLP1, which contains the conserved immune epitope nlp20 (ref. ^16^). However, rice does not naturally recognize this protein (Fig. 4a). To determine whether RLP23 can mediate MoNLP1 recognition in rice, we transiently expressed the *p35S::RLP23–GFP* construct in rice protoplasts. Upon MoNLP1 treatment, RLP23 expression triggered a significant accumulation of reactive oxygen species (ROS) (Fig. 4a). Moreover, MoNLP1-treated RLP23-expressing rice protoplasts showed significant upregulation of defense-related genes, including *OsWRKY70*, *OsPAL1*, *OsPR1a* and *OsMAPK6* (Fig. 4b). These results indicate that RLP23 enables MoNLP1 recognition and activates defense responses in rice.

To optimize RLP23 functionality in rice, we designed hybrid receptors RLP23/IC^OsRLP1^ and RLP23/CT^OsRLP1^ by fusing RLP23 with corresponding domains of rice OsRLP1, a key component of antiviral immunity in rice^17^. Compared with WT RLP23, expression of RLP23/IC^OsRLP1^ in rice protoplasts led to further increased ROS production and stronger defense gene induction upon MoNLP1 treatment, while further extending the modification to encompass the full C terminus amplified these immune responses (Fig. 4). However, fusion with the C terminus of tomato RLP EIX2 failed to boost RLP23 activity in rice, emphasizing the species-specific nature of CT domain compatibility for cross-species receptor function (Supplementary Fig. 9a). These findings underscore the potential of RLP engineering to improve disease resistance in monocot crops such as rice.

### Heterologous expression of *Arabidopsis* RLP23 enhances fungal resistance in poplar

**Fig. 5:**
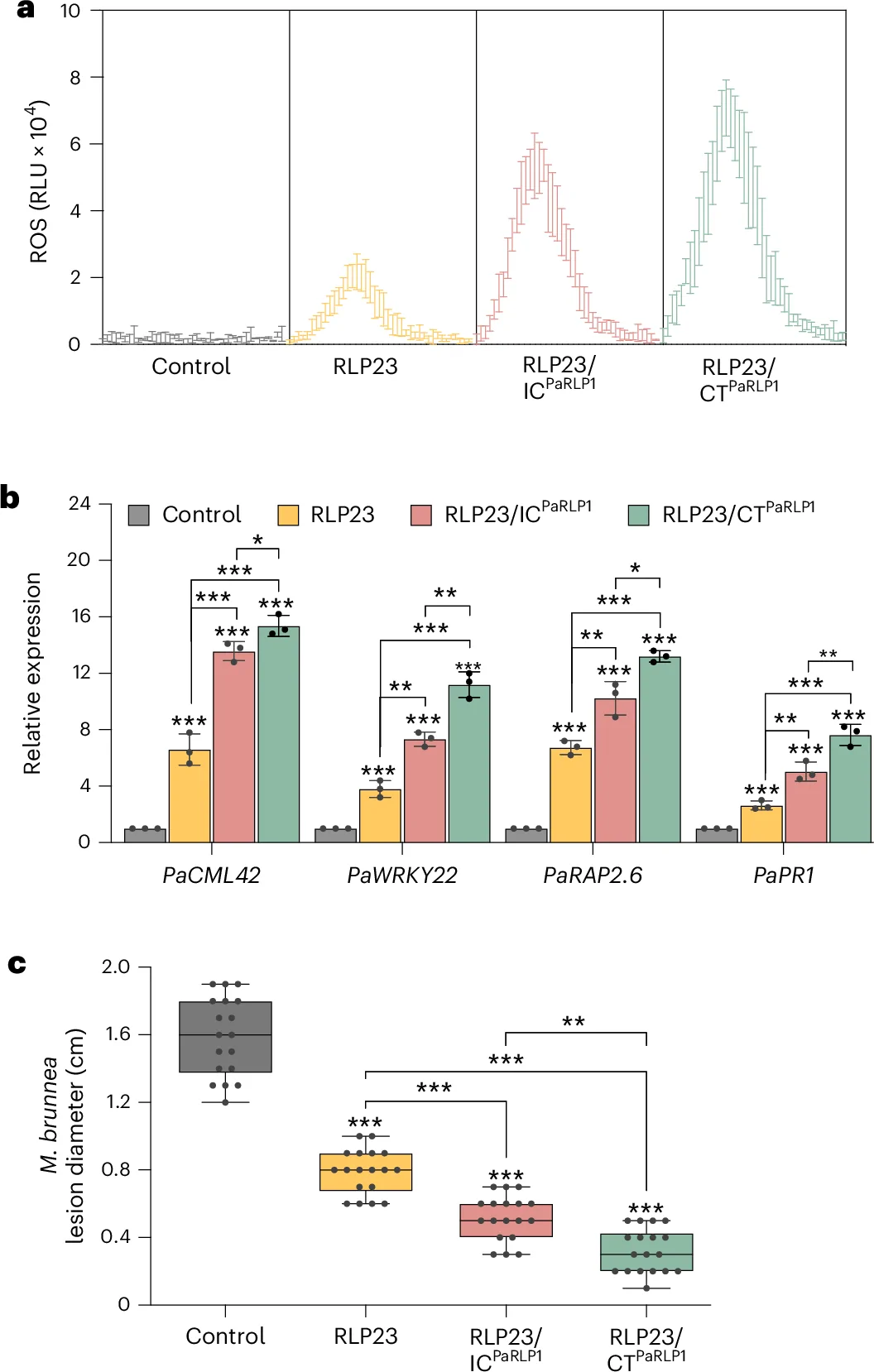
RLP23 and its hybrid form confer increased defense in poplar. **a**, ROS accumulation in poplar leaves 48 h after transient expression of GFP (control) or the indicated constructs, followed by treatment with 2 µM nlp24^MbNLP1^. ROS levels were recorded over 2 h and are presented as RLU. **b**, RT–qPCR analysis of poplar leaves expressing GFP (control), RLP23, RLP23/IC^PaRLP1^ or RLP23/CT^PaRLP1^ for 48 h, followed by treatment with 1 µM nlp24^MbNLP1^ for 24 h. Expression levels of the indicated genes were determined using gene-specific primers, normalized to *PaEF1α* transcript levels and presented relative to the control (set to 1). Data are presented as the mean ± s.d. (*n* = 3). **c**, Lesion diameter measured 3 days after inoculation with *M*. *brunnea* in poplar leaves transiently expressing GFP (control) or the indicated RLP–GFP fusion constructs. Data (*n* = 18) are shown as box plots (center line, median; bounds of box, the first and third quartiles; whiskers, 1.5 times the interquartile range; error bar, minima and maxima). All data were analyzed using a two-way ANOVA with Turkey’s test. Statistically significant differences from control plants are indicated (*P ≤ 0.05, **P ≤ 0.01 and ***P ≤ 0.001). Shown is one representative experiment of three with similar results. Source data

RLP23 is specific to the Brassicaceae family, while NLP proteins are widely conserved among fungal pathogens, including *Marssonina brunnea*, the causal agent of black spot disease in the economically important tree species poplar^18^. To explore the potential of cross-species RLP transfer for tree improvement, we used the NLP-type protein MbNLP1 from *M*. *brunnea*, which is recognized by RLP23 but not by poplar^18^ (Fig. 5a). MbNLP1 contains a conserved 24-aa immunogenic fragment (nlp24^MbNLP1^), which induces ROS accumulation and upregulates defense gene expression in poplar leaves transiently expressing RLP23 (Fig. 5a,b). Importantly, ectopic expression of RLP23 conferred fungal resistance, as evidenced by reduced disease symptoms in poplar leaves compared with GFP-expressing controls (Fig. 5c).

To further enhance RLP23 function in poplar, we engineered the chimeric receptors RLP23/IC^PaRLP1^ and RLP23/CT^PaRLP1^ by fusing the IC domain or the entire C terminus of poplar RLP1 with RLP23 (ref. ^19^). Similar to tomato and rice, poplar leaves expressing RLP23/CT^PaRLP1^ showed higher ROS accumulation and more pronounced defense gene expression in response to nlp24^MbNLP1^ compared with expression of WT RLP23 or the IC domain-swapped variant (Fig. 5a,b). These elevated immune responses translated into significantly enhanced antifungal resistance (Fig. 5c), demonstrating that RLP engineering can be effectively applied to tree breeding, offering a promising strategy for improving disease resistance in perennial crops.

## Discussion

Most resistance genes used in breeding programs originate from intracellular *NLR* receptors but pathogens rapidly evolve to evade NLR-mediated detection, thus undermining resistance^20^. In contrast, PRRs detect highly conserved molecular patterns, offering more durable, broad-spectrum defense. This makes PRR-based engineering a promising strategy for sustainable crop improvement.

Among PRRs, RLP23 and RLP30 stand out for their broad-spectrum pathogen recognition across bacterial, fungal and oomycete pathogens^8,9^. Notably, RLP23 retains functionality when transferred between species, conferring robust resistance in tomato against various pathogen classes. This aligns with previous findings in potato^8^, reenforcing RLP23 as a strong candidate for Solanaceae crop improvement. Remarkably, RLP23 also functions effectively in monocots such as rice and woody plants like poplar, enabling recognition of NLP proteins from pathogens such as *M*. *oryzae* (rice blast) and *M*. *brunnea* (poplar black spot disease). The ability of RLP23-expressing poplar leaves to resist *M*. *brunnea* infection further supports its cross-species functionality^18^.

The IC domain of RLPs, albeit short with 10–50 aa^21,22^, has a key role in heterologous expression systems. While dispensable for RLP23-mediated immunity in *Arabidopsis*, IC deletion weakens RLP23 function in *N*. *benthamiana*, emphasizing its role in facilitating immune signaling. Chimeric RLP23 receptors with IC domains from tomato RLPs enhance immune signaling in *N*. *benthamiana*, a strategy that also proved effective in rice and poplar. Similarly, replacing the IC domain of *Arabidopsis* RLK EFR with that of tomato RLP Cf-9 significantly boosted antibacterial immunity in Solanaceae species^23^, underscoring the applicability of RLP–RLK domain engineering to improve crop immunity.

Structural analyses show that the IC domains of tomato EIX2 (37 aa), Cf-9 (30 aa), OsRLP1 (41 aa) and PaRLP1 (43 aa) are longer than the RLP23 IC (19 aa), which may enhance interactions with SOBIR1. Removing the IC, TM and JM segments significantly reduced RLP function and its association with SOBIR1. Therefore, extending the engineering to the entire CT region optimizes RLP activity in heterologous systems. Similarly, *Arabidopsis* RLP1 becomes functional in tobacco only when fused to the C terminus of Solanaceous RLPs such as EIX2 (ref. ^24^). Coimmunoprecipitation assays confirmed that C-terminal engineering strengthens the RLP–SOBIR1 interaction without affecting ligand recognition mediated by the ectodomain.

However, RLP transfer across species is not always successful. For example, tomato Cf-9 does not confer Avr9 perception in *Arabidopsis* (Supplementary Fig. 10a), likely because of incompatibility with *Arabidopsis* coreceptors. Notably, native SlSOBIR1 is essential for Cf-4 stabilization^25^. Similarly, replacing the Cf-9 C terminus with that of RLP23 or RLP30 did not restore its function (Supplementary Fig. 10a), suggesting that additional species-specific factors, such as glycosylation patterns, may affect RLP folding and stability. Moreover, the Cf-9 CT region disrupts RLP23 function in *Arabidopsis*, highlighting the complexities of cross-species RLP adaptation (Supplementary Fig. 10b).

Importantly, engineered ‘super’ tomato lines exhibit broad-spectrum immunity to bacterial, fungal and oomycete pathogens without compromising growth or yield—a major milestone in disease resistance breeding. The enhanced disease resistance conferred by domain engineering is likely driven by multiple mechanisms. First, the tomato RLP C terminus improves the cross-species compatibility of RLP23, facilitating its functional integration into the tomato immune system. Additionally, the engineered RLP23 receptors exhibit stronger ligand-triggered immune responses in heterologous expression systems and modification of the C terminus may further reinforce the general synergistic interaction between pattern-triggered immunity and ETI during infection, as previously reported for nlp20-induced mutual potentiation^26^. Notably, the expression of chimeric RLP23 receptors confers nlp20-induced cell death in tobacco plants, suggesting that PRR engineering could approach or match the resistance levels obtained with NLRs. In summary, our findings highlight the essential role of the CT domains in RLP-mediated immunity and its potential to enhance disease resistance across diverse plant species. RLP23 engineering achieves broad-spectrum resistance while maintaining plant growth and productivity. Unlike NLR engineering, which focuses on expanding effector recognition, RLP modifications confer robust immunity to a wide range of pathogens. RLP23’s ability to function independently of additional adaptors or helpers simplifies resistance breeding. The versatility of RLP23 across monocots, dicots and woody plants underscores its potential for enhancing crop resilience. Future studies should focus on advancing RLP engineering by optimizing ectodomains for broader pathogen recognition, exploring RLP–NLR coexpression strategies to boost immunity, dissecting the underlying molecular mechanisms that strengthen heterologous resistance and evaluating the long-term stability and field performance of transgenic crops to strengthen global food security.

## Methods

### Plant materials and growth conditions

*A*. *thaliana* WT (Col-0), mutant and transgenic plants (Supplementary Table 1) were grown in soil for 7–8 weeks within a growth chamber, maintaining short-day conditions (8-h photoperiod, 22 °C temperature and 40–60% humidity). *Solanum* *lycopersicum* Moneymaker WT and transgenic plants (Supplementary Table 1), *N*. *benthamiana*, rice (*Oryza* *sativa* subsp. *japonica*) and poplar (*Populus* *bolleana*) plants were cultivated in a greenhouse at 23 °C under long-day conditions, with 16 h of light exposure and humidity maintained at 60–70%.

### Peptides and elicitors

Synthetic peptides nlp20, nlp24 and nlp24^MbNLP1^ (GenScript) and elicitors EIX (xylanase from *Trichoderma* *viride*; X3876, Sigma-Aldrich) and MoNLP1 (expressed in *Pichia* *pastoris)* were prepared as 10 mM stock solutions in 100 % dimethyl sulfoxide and diluted in water to the desired concentration before use. GFP and Avr9 proteins were expressed in *N*. *tabacum* leaves^27^ and the recombinant SCP was expressed in *P*. *pastoris* KM71H and subsequently purified^9^.

### Construction of RLP chimeras and mutations

IC domain swap constructs were generated using the Gibson assembly master mix kit (New England Biolabs). Coding sequences for *RLP23*, *RLP30*, *Cf-9*, *EIX2*, *OsRLP1* and *PaRLP1* (Supplementary Table 4) were cloned into pDONR207, pCR8 (Thermo Fisher Scientific) or the pLOCG vector. Using these plasmids as templates, fragments of the RLP IC domain and the entire C terminus containing overlapping regions were amplified and assembled with SpeI-digested pLOCG to produce chimeric constructs with C-terminal GFP fusions. Primers (Eurofins Genomics) are listed in Supplementary Table 2. Putative phosphorylation sites in RLP30 (RLP30^4mut^) and RLP23 (RLP23^2mut^) were mutated using overlapping PCR and primers listed in Supplementary Table 2. Epitope tagging of nonchimeric constructs was achieved by recombination of entry clones using Gateway cloning (Thermo Fisher Scientific) into pGWB5 (C-terminal GFP tag) or pGWB14 (C-terminal HA tag). Constructs for NbSOBIR1–HA and SlSOBIR1–HA were described previously^10^.

### Generation of transgenic plants

For stable integration of RLP constructs into *Arabidopsis* WT Col-0 or *rlp23*-1 mutants or tomato Moneymaker, *Agrobacterium* *tumefaciens* GV3101 strains carrying the constructs were grown in Luria–Bertani (LB) medium with appropriate antibiotics. For *Arabidopsis* transformation, bacterial cultures were harvested and resuspended in 5% (w/v) sucrose with 0.02 % (v/v) Silwet to an optical density (OD) of 0.8; inflorescences of 6–8-week-old *Arabidopsis* plants were dipped for 1.5 min (ref. ^9^). Then, 0.2% (v/v) BASTA was used for T1 selection. For tomato transformation, bacterial cells were resuspended in 10 mM MgCl_2_ and leaf pieces were incubated in the suspension for 3 min before transfer to Murashige–Skoog (MS) medium with 2% (w/v) sucrose for 48 h in the dark. Transgenic calli were selected on MS medium with appropriate antibiotics and then transgenic plants were moved from sterile culture to soil in a greenhouse under long-day conditions. Protein expression was confirmed by western blot analyses with anti-HA and anti-GFP antibodies (Thermo Fisher Scientific). To generate *NbRE02*-knockout mutants, gene-specific sgRNAs (sgRNA1: 5′-CTACGGTATCCTCTCGTCAT-3′; sgRNA2: 5′-CTAGACGGGAGTTCAGGCTA-3′) were designed on the basis of the previously reported coding sequence of *NbRE02* in *N*. *benthamiana* using the online tool CCTop (https://cctop.cos.uni-heidelberg.de/). The pHEE401 vector carrying the gRNA sequences was introduced into *N*. *benthamiana* callus by *Agrobacterium*-mediated transformation. To identify CRISPR–Cas9-edited mutants, genomic DNA was extracted from mutant seedlings and the targeted regions were verified by sequencing.

### Transient expression in plants

*A*. *tumefaciens* GV3101 carrying desired constructs was cultured overnight at 28 °C in LB medium with selective antibiotics, collected by centrifugation and resuspended in a solution of 10 mM MgCl_2_, 10 mM MES pH 5.7 and 150 μM acetosyringone. The cultures were adjusted to an OD_600_ of 0.5, incubated for 2 h at room temperature and then infiltrated into 4-week-old *N*. *benthamiana* or poplar leaves. Leaf samples were collected 48 h after infiltration for ethylene production, ROS burst, reverse transcription (RT)–qPCR and infection assays.

For transient expression in rice protoplasts, PEG-mediated transfection was performed following established protocols^28^. Briefly, 5–10 μg of plasmid DNA was mixed with 100 μl of protoplast suspension. MoNLP1 was further added to the protoplasts at a desired final concentration after 2 days.

### In vivo crosslinking and immunoprecipitation assays

For in vivo crosslinking, *N*. *benthamiana* leaves expressing RLP23–GFP were infiltrated with 50 nM nlp24-bio peptide, with or without 100 µM unlabeled nlp24 peptide as competitor. Then, 5 min after infiltration, 2 mM ethylene glycol bis(succinimidyl succinate) was introduced for peptide–receptor crosslinking^8^. After 20 min, leaf samples were collected and immediately frozen in liquid nitrogen. For immunoprecipitation assays, constructs were transiently expressed for 48 h in *N*. *benthamiana* leaves. Total protein was extracted from approximately 300 mg of leaf sample and subjected to immunoprecipitation using GFP-Trap agarose beads (ChromoTek). Proteins were detected by immunoblotting with epitope-tag-specific primary antibodies (anti-GFP monoclonal antibody (GF28R), Thermo Fisher Scientific, MA5-15256; anti-HA monoclonal antibody (RM305), Thermo Fisher Scientific, MA5-27915; anti-biotin monoclonal antibody (BN-34), Sigma, B7653), followed by horseradish peroxidase (HRP)-conjugated secondary antibodies and enhanced chemiluminescence (Cytiva)^9^.

### Plant immune responses

Ethylene production was triggered in three leaf pieces floating on 0.5 ml of 20 mM MES buffer pH 5.7 with the indicated elicitors. After 4 h of incubation, 1 ml of air was sampled from sealed assay tubes and ethylene levels were measured using gas chromatography (GC-14A, Shimadzu)^29^. For detection of the ROS burst, one leaf piece per well was placed in a 96-well plate (Greiner BioOne) containing 100 μl of a solution with 20 μM luminol derivative L-012 (Wako Pure Chemical Industries) and 20 ng ml^−1^ HRP (Applichem). Luminescence was recorded at 2-min intervals, first for background and then for up to 60 min after elicitor or mock treatment, using a Mithras LB 940 luminometer (Berthold Technologies)^9^. For RT–qPCR analysis, total RNA from plant or protoplast samples was isolated using the RNeasy plant mini kit (Qiagen) and used for gene expression analysis with the gene-specific primers (Eurofins Genomics) listed in Supplementary Tables 3 and 4.

### Infection assay

For bacterial infection, leaves of 6-week-old tomato plants were infiltrated with *Pst* DC3000 at a final concentration of 10^5^ colony-forming units (CFU) per ml and collected 3 days after inoculation^9^. For fungal infection, spores of *B*. *cinerea* B05.10 were diluted to a final concentration of 10^6^ spores per ml and 5-μl drops were applied to tomato leaves. Lesion sizes were measured 2 days after inoculation by averaging lesion diameters. Poplar leaves transiently expressing different forms of RLP23 were inoculated with a conidial suspension of *M*. *brunnea* f. sp. *monogermtubi* (10^6^ conidia per ml)^18^. Inoculated leaves were placed on 1.5 % water agar in 9-cm Petri dishes and incubated at 25 °C. Infection diameter was recorded 5 days after inoculation. For oomycete infection, tomato leaves were inoculated with a 10-µl drop of *P*. *infestans* spore suspension (10^6^ spores per ml). Lesion areas were assessed 4 days after inoculation^30^.

### Data analysis

No statistical methods were used to predetermine sample size. The experiments were not randomized. The investigators were not blinded to allocation during experiments and outcome assessment. Data were plotted and analyzed using GraphPad Prism (version 10.2) or Microsoft Office Excel. Data were presented as the mean ± s.d. or as box-and-whisker plots in which the center line indicates the median, the bounds of the box indicate the 25th and 75th percentiles and whiskers indicate 1.5 times the interquartile range between the 25th and 75th percentiles.

### Reporting summary

Further information on research design is available in the Nature Portfolio Reporting Summary linked to this article.

## Online content

Any methods, additional references, Nature Portfolio reporting summaries, source data, extended data, supplementary information, acknowledgements, peer review information; details of author contributions and competing interests; and statements of data and code availability are available at https://doi.org/10.1038/s41587-025-02858-8.

## Supplementary information

Supplementary Information Supplementary Figs. 1–10, Tables 1–4 and unprocessed western blots for supplementary figures.

Reporting Summary

Supplementary Data 1 Statistical source data for supplementary figures.

## Source data

Source Data Figs. 1, 2, 4 and 5 Statistical source data.

Source Data Fig. 3 Unprocessed western blots.

## Data Availability

All data are available in the main text, extended data or Supplementary Information. Source data are provided with this paper.
