# Supplementary material for: Engineered pattern recognition receptors enhance broad-spectrum plant resistance
Source: Nat Biotechnol. 2025 Oct 13;44(10):1715–21. doi: 10.1038/s41587-025-02858-8 (PMC13654806; doi:10.1038/s41587-025-02858-8)
Supplement: Supplementary file 1 [file 41587_2025_2858_MOESM1_ESM.pdf]

nature biotechnology

Supplementary information

https://doi.org/10.1038/s41587-025-02858-8

Engineered pattern recognition receptors enhance broad-spectrum plant resistance

In the format provided by the authors and unedited

Table of Contents

Supplementary Figures (Page 2 – 11)

Figure S1: Growth, development, and morphology of transgenic tomato plants stably expressing RLP23 or RLP23 chimeras are indistinguishable from wild-type plants

Figure S2: Structural characterization of RLP

Figure S3: Deletion of the Intracellular domain of RLPs doesn't affect its immune activity in Arabidopsis but reduces functionality in *N. benthamiana*

Figure S4: Deletion of the C-terminus of RLPs reduces their immune activity in *N. benthamiana*

Figure S5: C-terminal domain swapping enhances RLP30-mediated immunity in *N. benthamiana*

Figure S6: C-terminal swapping alters tomato RLP functionality in *N. benthamiana*

Figure S7: Expression of RLP23/CTEIX2 in tomato significantly enhances nlp20-induced expression of defense-related genes

Figure S8: Replacement of the C-terminus of RLP23 alters its interaction with the adaptor SOBIR1.

Figure S9: The tomato EIX2 C-terminus does not enhance RLP23-mediated immunity in rice or poplar.

Figure S10: Cf-9 or its chimeric versions are not functional in Arabidopsis

Supplementary Tables (Page 12 – 16)

Supplementary Table 1. Plants used in this study

Supplementary Table 2. Primers used for cloning

Supplementary Table 3. Primers used for RT-PCR

Supplementary Table 4. Accession numbers of genes used in this study

Uncropped Western Blots (Page 17)

Unprocessed Western Blots for Figures S1b and S3g,h,i,j

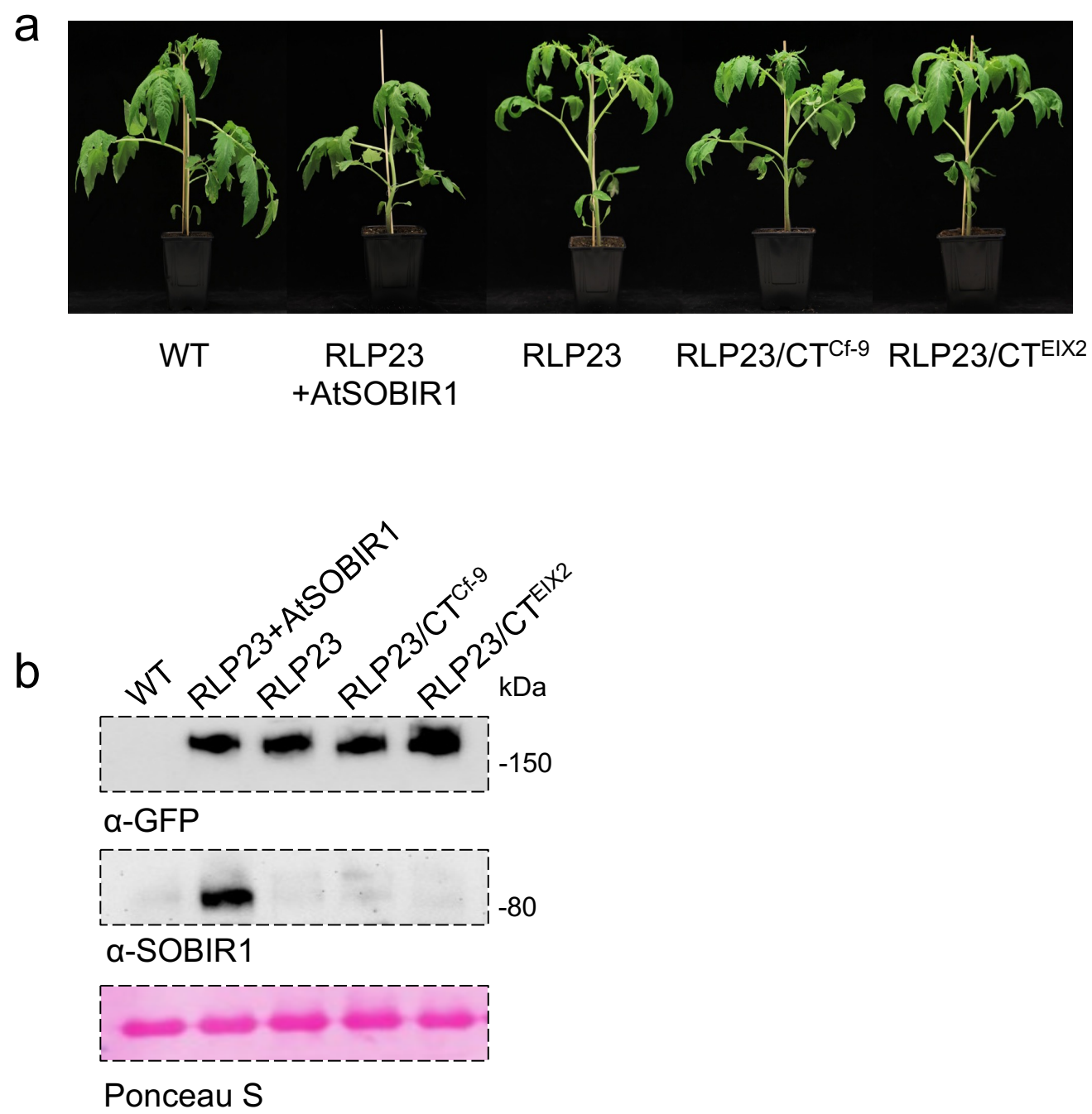

Figure S1: Growth, development, and morphology of transgenic tomato plants stably expressing RLP23 or RLP23 chimeras are indistinguishable from wild-type plants

a, Photographs of four-week-old wild-type (WT) plants or different transgenic lines stably expressing RLP23-GFP with or without AtSOBIR1-HA, RLP23/CT$^{Cf\text{-}9}$-GFP or RLP23/CT^{EIX2}-GFP. **b,** Western Blot analysis on tomato protein extracts from transgenic lines shown in (a) using an anti-GFP and anti-SOBIR1 antibody, equal loading was verified by staining of the membrane with Ponceau S Red.

a

b

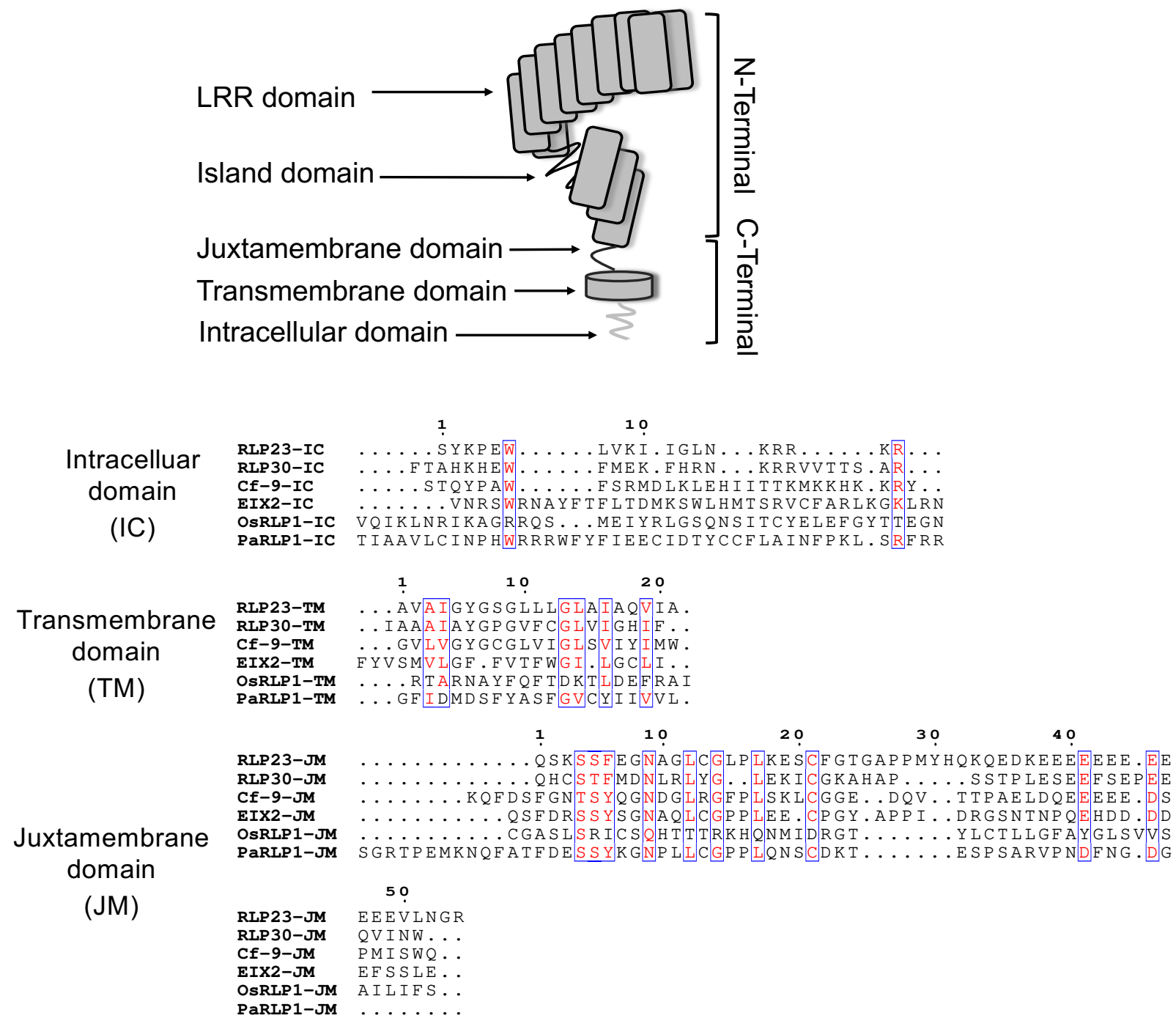

c

	Number of LRR domains	Intracellular domain (aa)	Transmembrane domain (aa)	Juxtamembrane domain (aa)	Full length (aa)
RLP23	27	19	21	64	890
RLP30	22	26	21	35	786
Cf-9	26	30	21	53	863
EIX2	31	37	21	50	1020
OsRLP1	26	41	21	48	980
PaRLP1	31	43	21	52	1039

Figure S2: Structural characterization of RLP

a, Schematic representation of the structure of an RLP. **b,** Sequence alignment of the C-terminal domains of all RLPs involved in this study. Boxes/red color indicate the most conserved amino acid residues. **c,** Comparison of domain lengths at the C-terminal region of various RLPs.

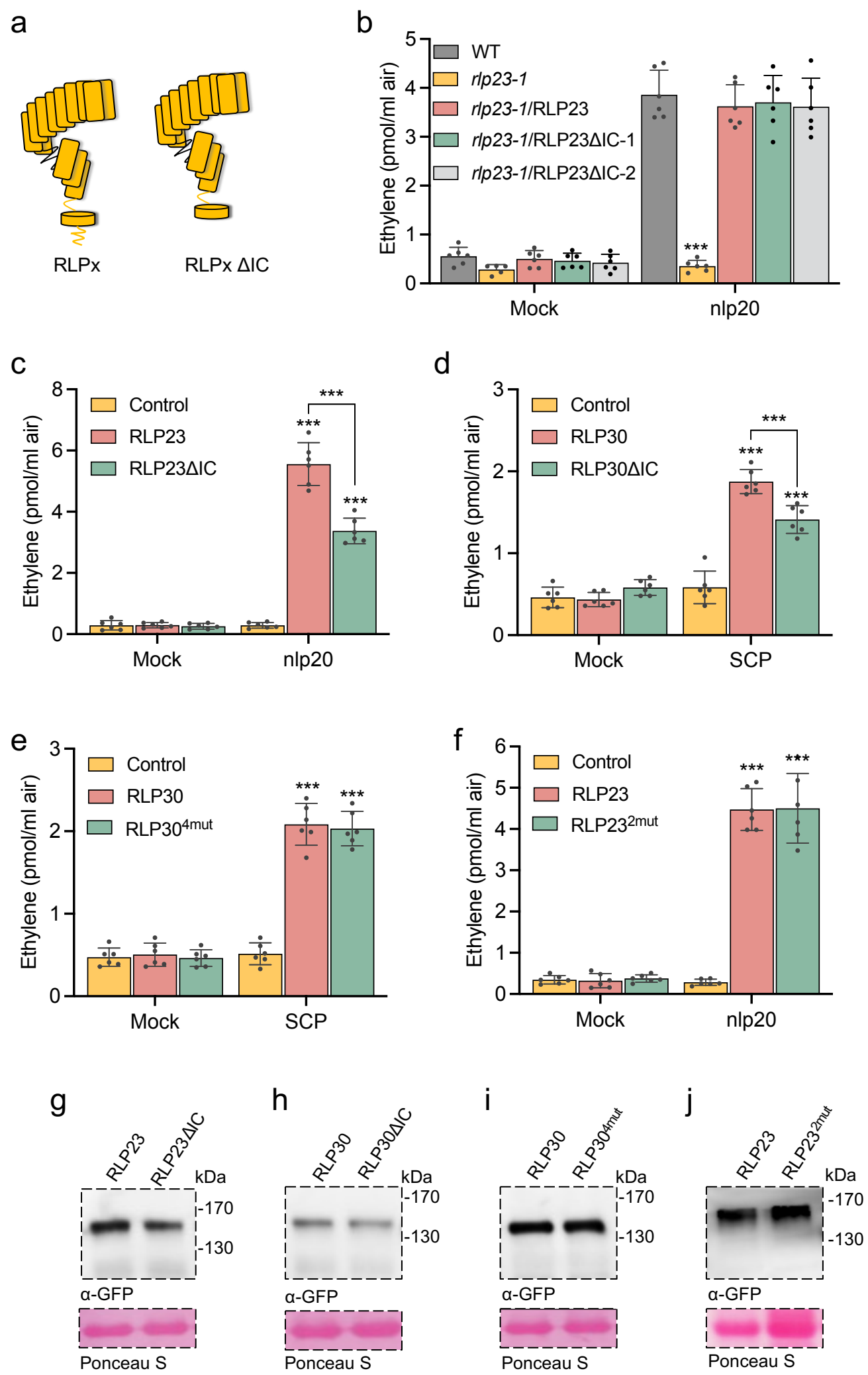

Figure S3: Deletion of the Intracellular domain of RLPs doesn't affect its immune activity in Arabidopsis but reduces functionality in *N. benthamiana*

a, Schematic representation of the structure of RLP-IC domain deletion. **b,** Ethylene accumulation in wild-type (WT), *rlp23-1* and transgenic *rlp23-1* Arabidopsis plants stably expressing RLP23 or a RLP23 variant lacking the intracellular domain (RLP23ΔIC, 2 independent lines) after 4 h treatment with water (mock) or 1 µM nlp20. **c,d,** Ethylene accumulation in *N. benthamiana* transiently expressing GFP (Control), RLP23 or RLP23ΔIC after treatment for 4 h with water (mock) or 2 µM nlp20. **d,e,** Ethylene accumulation induced by 1 µM SCP in *RE02*-knockout *N. benthamiana* leaves transiently expressing GFP (Control), RLP30, an RLP30 variant lacking the intracellular domain (RLP30ΔIC, **d**) or a quadruple mutant in which the putative C-terminal phosphorylation sites T761, T783, T784, and S785 were replaced by alanine (4mut, **e**). **f,** Ethylene accumulation induced by 2 µM nlp20 in *N. benthamiana* leaves transiently expressing GFP (Control), RLP23, or RLP23^{2mut} in which the putative C-terminal phosphorylation sites S872 and Y873 were mutated. **g,h,i,j,** Western Blot analysis on *N. benthamiana* protein extracts from leaves shown in c-f, using an anti-GFP antibody. Equal loading was verified by staining of the membranes with Ponceau S Red. Bars represent means ± s.d. of six independent experiments for **b-f**. Data were analyzed by Two-way ANOVA with Tukey's test. Statistically significant differences from WT or control are indicated (***P ≤ 0.001). Exact P values are provided in Supplementary data. All experiments were repeated at least three times with similar results.

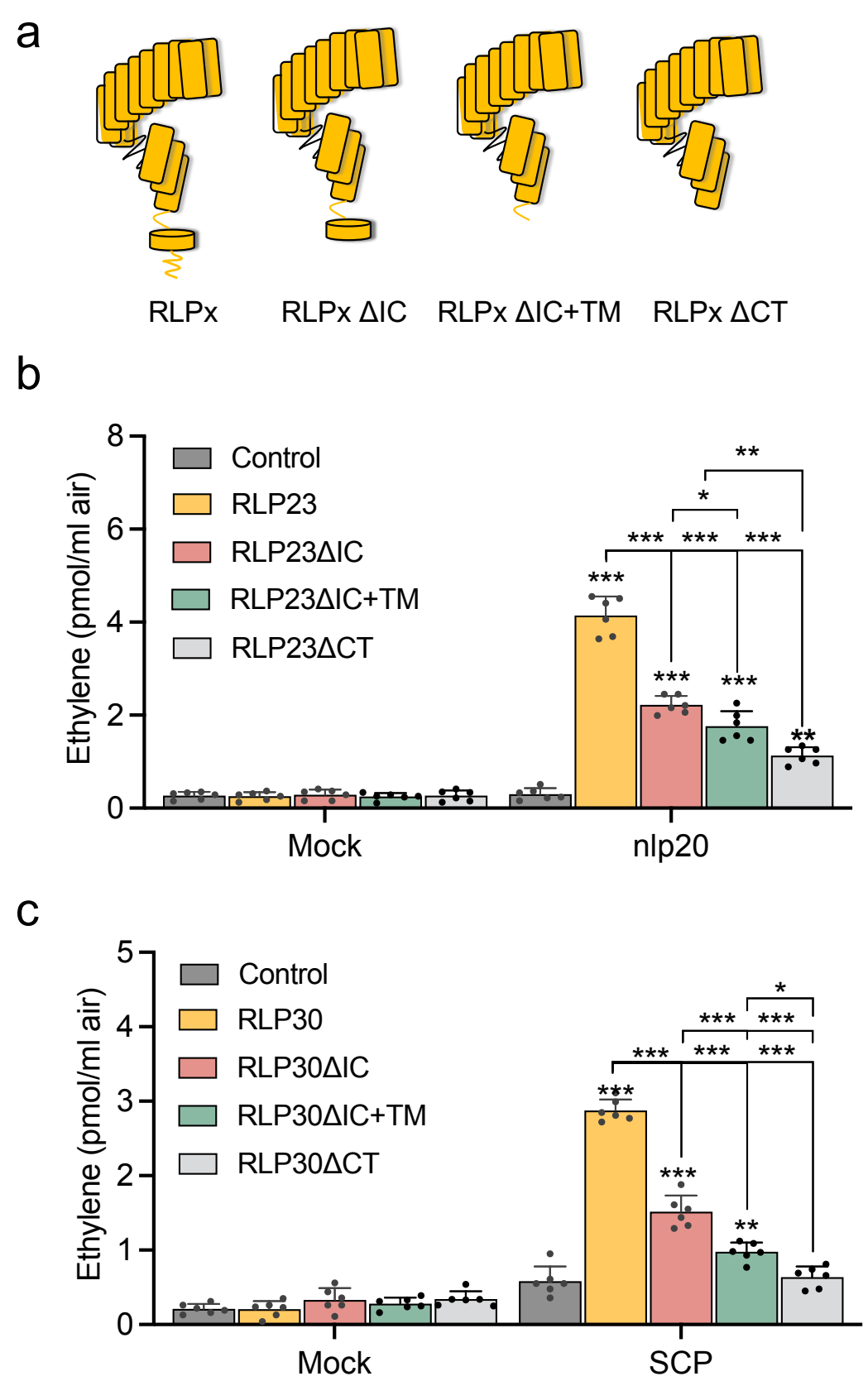

Figure S4: Deletion of the C-terminus of RLPs reduces their immune activity in *N. benthamiana*
a, Schematic representation of C-terminal truncation of RLPs. **b,** Ethylene accumulation in *N. benthamiana* transiently expressing GFP (Control), RLP23, or RLP23 variants lacking the intracellular domain (RLP23ΔIC), the intracellular domain and the transmembrane domain (RLP23ΔIC + TM) or the full C-terminus (RLP23ΔCT) after treatment for 4 h with water (mock) or 2 µM nlp20. **c,** Ethylene accumulation induced by 1 µM SCP in *RE02*-knockout *N. benthamiana* leaves transiently expressing GFP (Control), RLP30, or indicated RLP30 variants. Bars represent means ± s.d. of six independent experiments for **b,c**. Data were analyzed by Two-way ANOVA with Tukey's test. Statistically significant differences from the control are indicated (***P ≤ 0.001). Exact P values are provided in Supplementary data. All experiments were repeated at least three times with similar results.

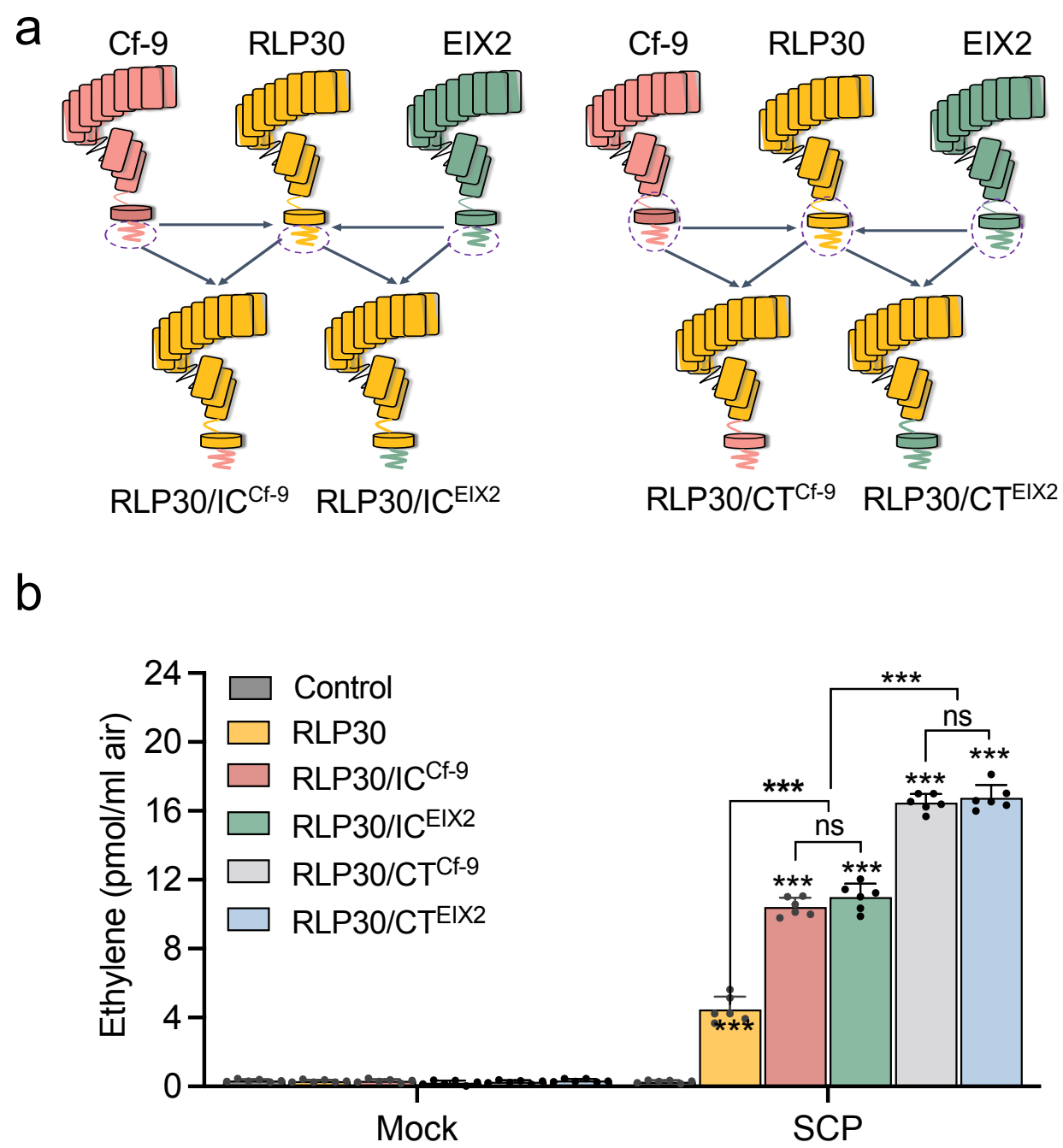

Figure S5: C-terminal domain swapping enhances RLP30-mediated immunity in *N. benthamiana*
a, Schematic representation of RLP30, Cf-9, and EIX2, along with their chimeric variants with different C-terminal domains swaps. **b,** Ethylene accumulation in *N. benthamiana* leaves transiently expressing GFP (Control), RLP30-GFP, RLP30/IC^{Cf-9}-GFP, RLP30/IC^{EIX2}-GFP, RLP30/CT^{Cf-9}-GFP, or RLP30/CT^{EIX2}-GFP, measured 4 hours after treatment with water (mock) or 1 µM SCP. Bars represent means ± s.d. of six independent experiments. Data were analyzed by Two-way ANOVA with Tukey's test. Statistically significant differences from Control are indicated (***P ≤ 0.001). Exact P values are provided in Source data. All experiments were repeated at least three times with similar results.

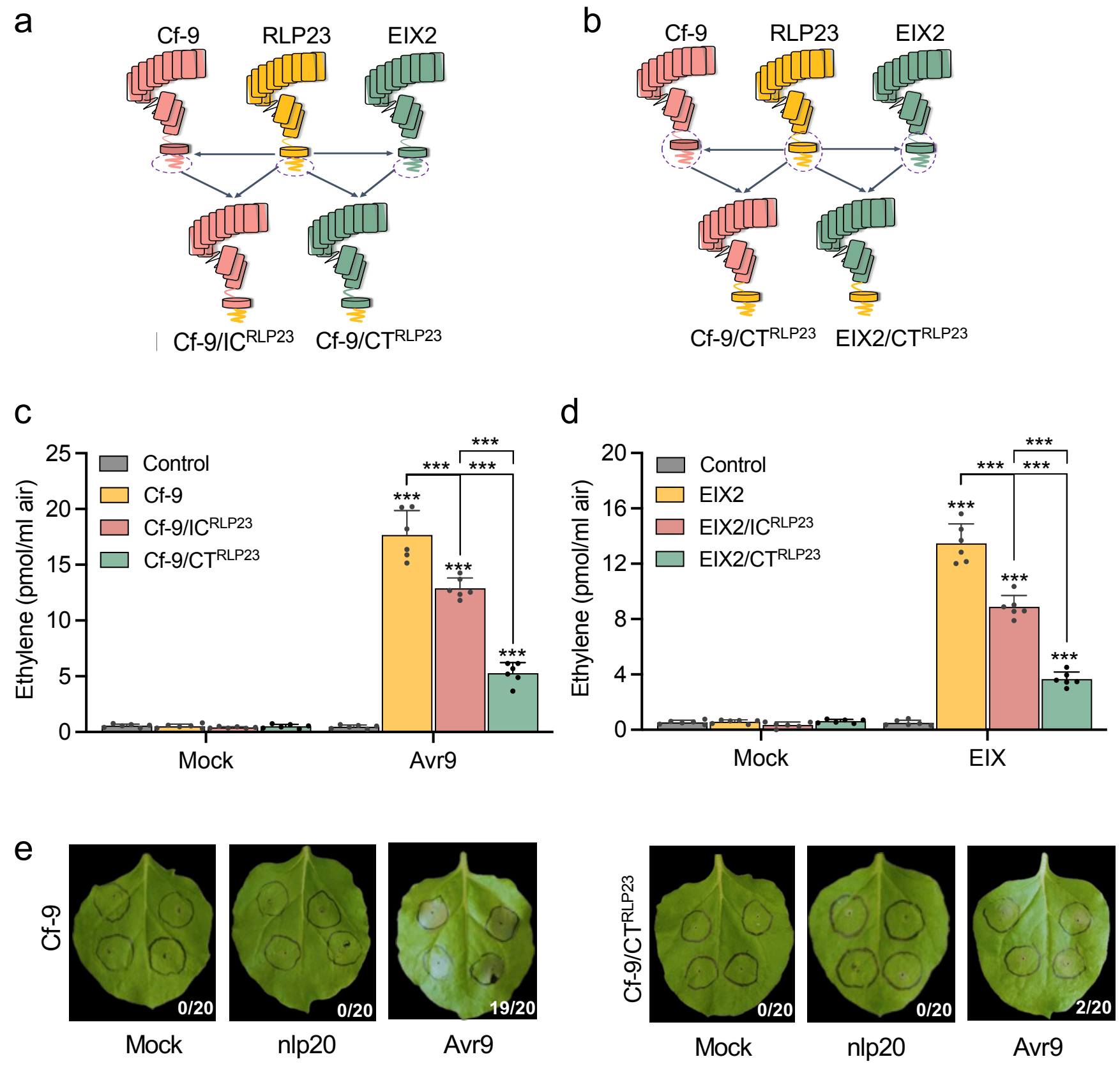

Figure S6: C-terminal swapping alters tomato RLP functionality in *N. benthamiana*

a,b Schematic representation of RLP23, Cf-9, and EIX2, along with their chimeric variants featuring reciprocal C-terminal swaps. **c,** Ethylene accumulation in *N. benthamiana* leaves expressing GFP (Control), Cf-9-GFP, Cf-9/IC^{RLP23} or Cf-9/CT^{RLP23}-GFP, assessed 4 hours after treatment with 5 µl of *N. tabacum* apoplastic fluid with Avr9 or without (mock). **d,** Ethylene accumulation in *N. benthamiana* leaves expressing GFP (Control) or indicated RLP-GFP constructs, measured 4 hours after treatment with water (mock) or 1 µM EIX. **d,** Cell death development in *N. benthamiana* leaves transiently expressing Cf-9 or Cf-9/CT^{RLP23}, 72 hours after infiltration with water (mock), 20 µM nlp20, or 100 µl apoplastic fluid containing Avr9. The number of necrotic leaves is presented relative to the total number of infiltrated leaves. Bars represent means ± s.d. of six independent experiments for **c,d**. Data were analyzed by Two-way ANOVA with Tukey's test. Statistically significant differences from Control are indicated (***P ≤ 0.001). Exact P values are provided in Source data. All experiments were repeated at least three times with similar results.

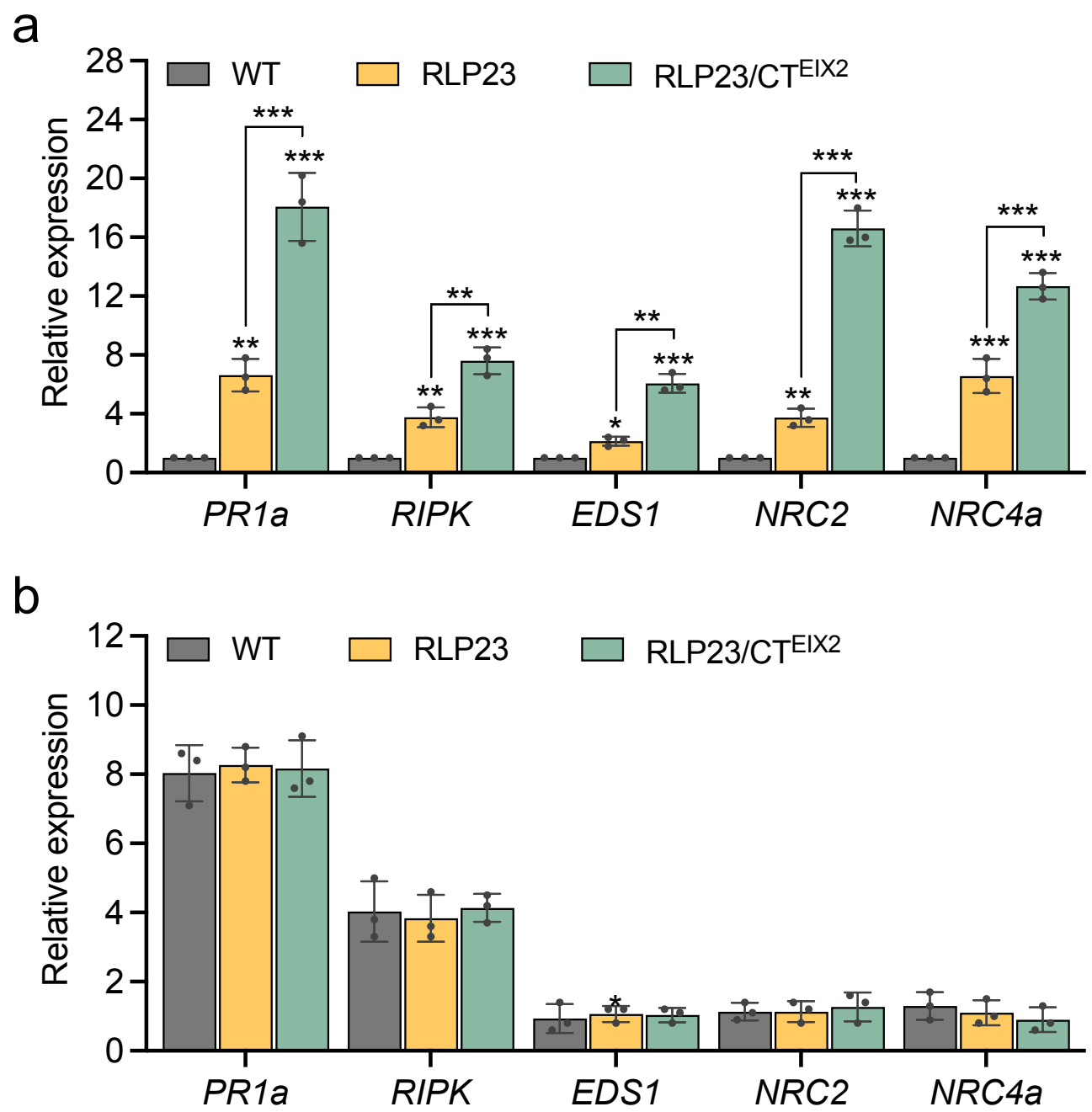

Figure S7: Expression of RLP23/CT^{EIX2} in tomato significantly enhances nlp20-induced expression of defense-related genes

a,b, RT-qPCR analysis in wild-type (WT) and transgenic tomato plants stably expressing RLP23-GFP or RLP23/CT^{EIX2}-GFP after 2 µM nlp20 (**a**) or 0.5 µM flg22 (**b**) treatment for 24 h using gene specific primers for the indicated genes. Expression of marker genes was normalized to the levels of *SlEF1α* transcript and are presented relative to the wild-type control which was set to 1(**a**). Bars represent means ± s.d. of six independent experiments. Data were analyzed by Two-way ANOVA with Tukey's test. Statistically significant differences from the control are indicated (*P ≤ 0.05, **P ≤ 0.01, ***P ≤ 0.001). Exact P values are provided in Supplementary data. All experiments were repeated at least three times with similar results.

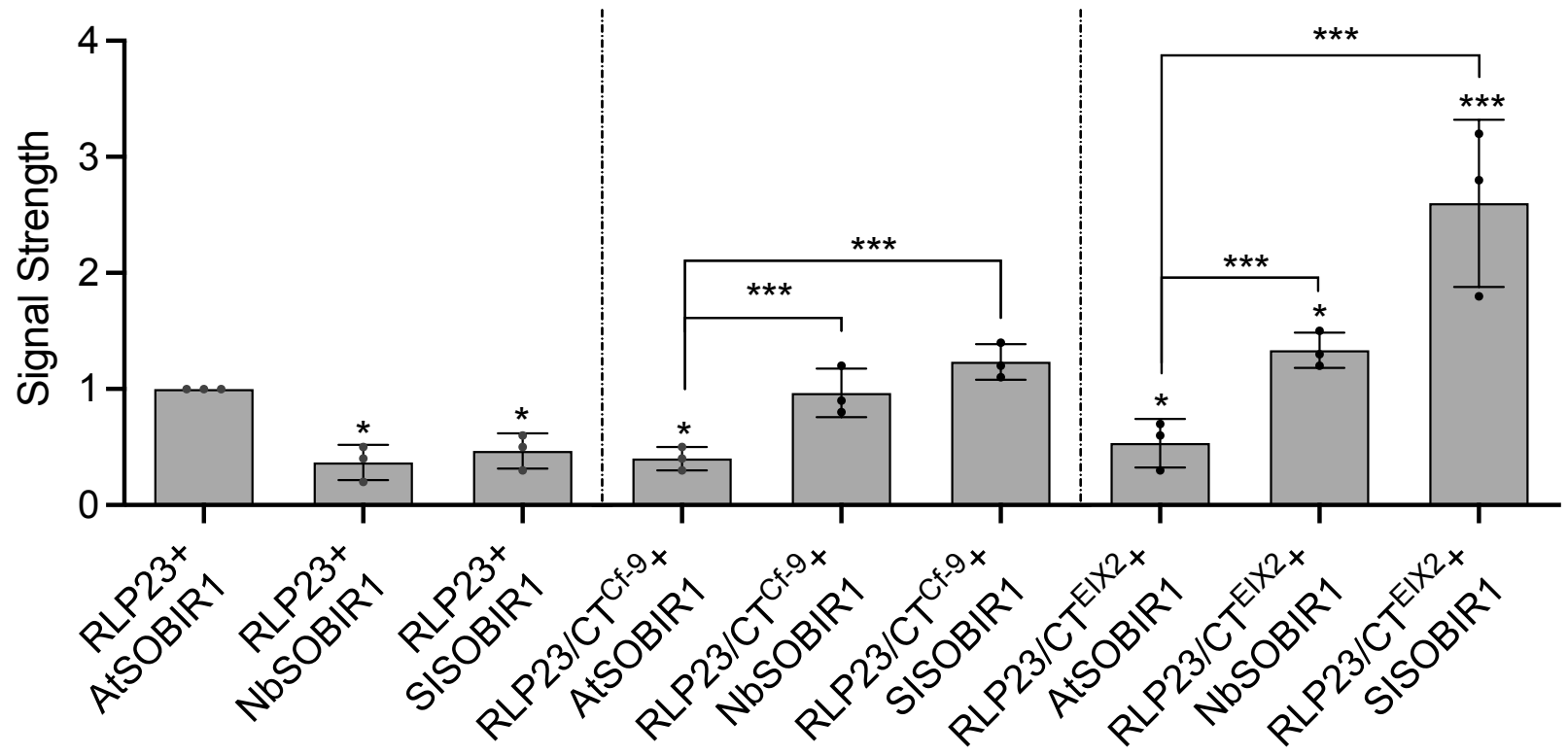

Figure S8: Replacement of the C-terminus of RLP23 alters its interaction with the adaptor SOBIR1. Co-immunoprecipitation assay in *N. benthamiana* leaves co-expressing GFP-tagged RLP23 variants with AtSOBIR1-HA, NbSOBIR1-HA, or SlSOBIR1-HA. Leaf protein extracts (Input) were subjected to co-immunoprecipitation with GFP beads (IP:GFP) and immunoblotting using tag-specific antibodies. Protein levels were quantified relative to AtSOBIR1-HA precipitated by RLP23-GFP, set as 1. Bars represent means ± s.d. of three independent experiments. Data were analyzed by Two-way ANOVA with Tukey's test. Statistically significant differences from the interaction of RLP23 + AtSOBIR1 are indicated (*P ≤ 0.05, **P ≤ 0.01, ***P ≤ 0.001). Exact P values are provided in Supplementary data. All experiments were repeated at least three times with similar results.

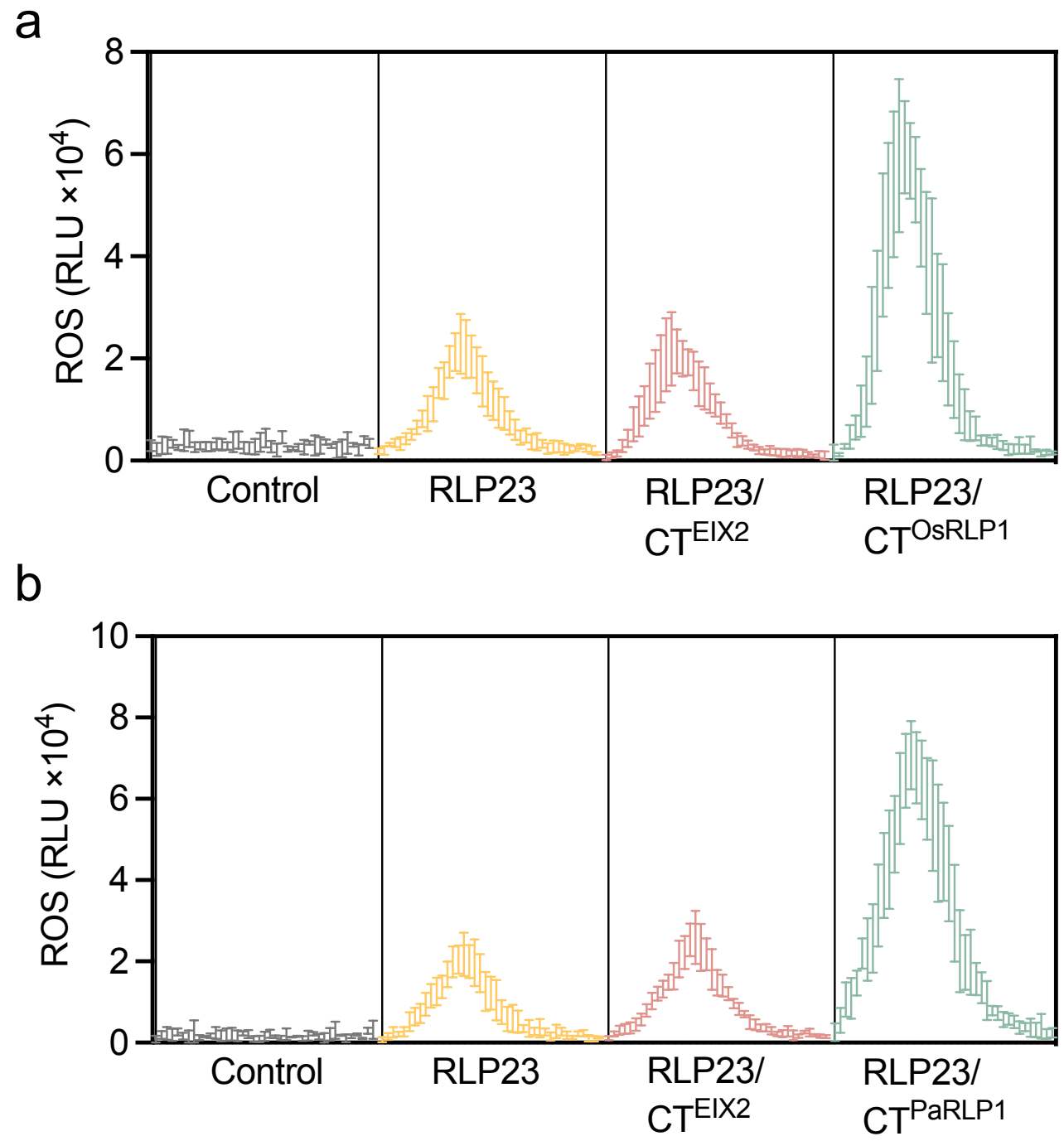

Figure S9: The tomato EIX2 C-terminus does not enhance RLP23-mediated immunity in rice or poplar.

a, Reactive oxygen species (ROS) production in rice protoplasts expressing GFP (Control), RLP23-GFP, RLP23/CT^{EIX2}-GFP or RLP23/CT^{OsRLP1}-GFP, following treatment with 5 μM purified MoNLP1. Data are presented as relative light units (RLU) over 2 hours. **b,** ROS production in poplar leaves transiently expressing GFP (Control) or the indicated constructs for 48 hours. ROS accumulation was measured for 2 hours following treatment with 2 μM $nlp24^{MbNLP1}$ and is given in relative light units (RLU). Each experiment was performed three times with similar results.

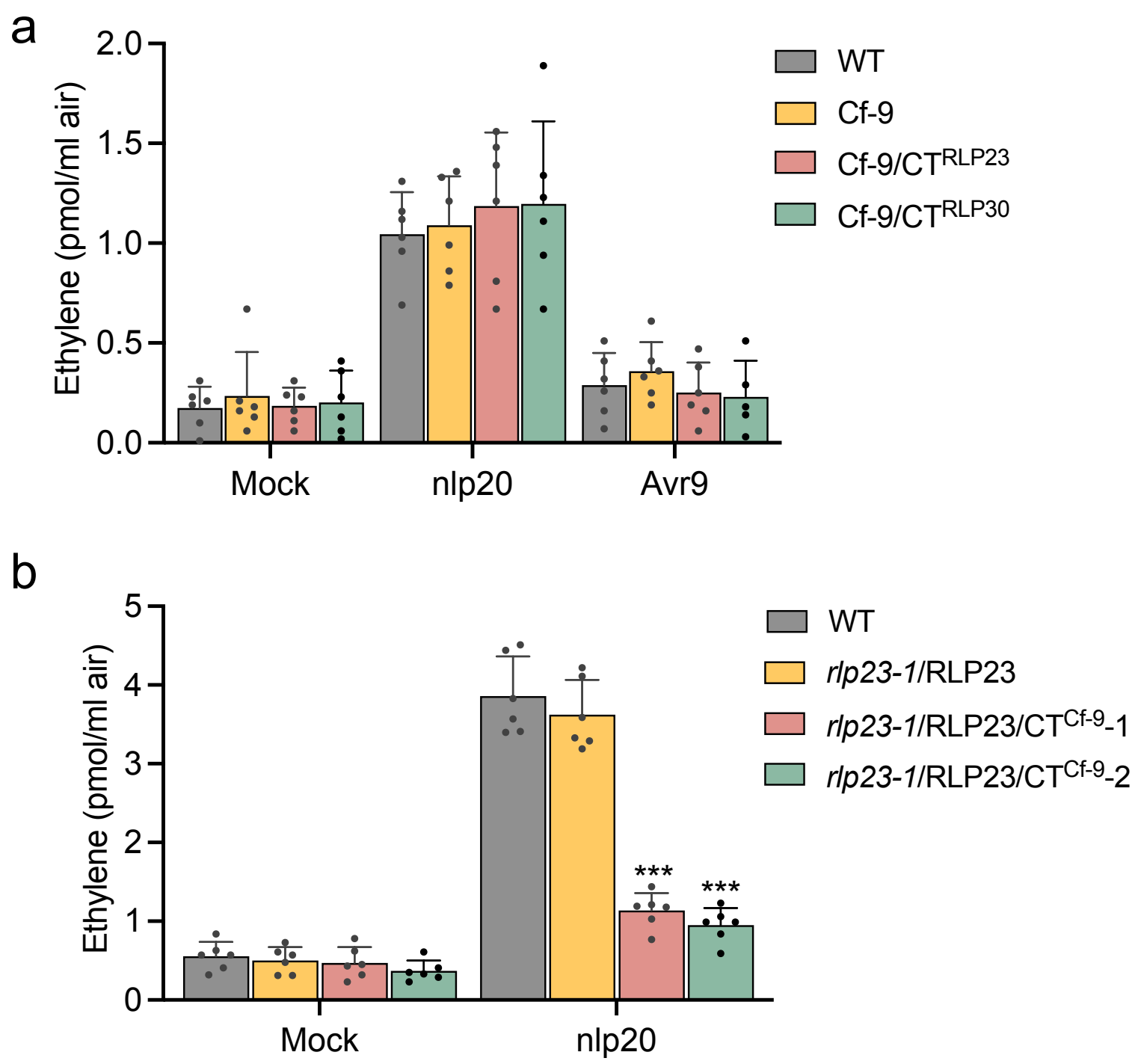

Figure S10: Cf-9 or its chimeric versions are not functional in Arabidopsis

a, Ethylene accumulation in wild-type (WT) Arabidopsis plants or transgenic lines harboring constructs for the expression of Cf-9, Cf-9/CT^{RLP23} or Cf-9/CT^{RLP30} after 4 h treatment with 5 µl apoplastic fluid containing GFP (Mock) or Avr9, or 1 µM nlp20. **b,** Ethylene accumulation in wild-type (WT) and transgenic *rlp23-1* lines stably expressing RLP23 or RLP23/$CT^{Cf\text{-}9}$ after 4 h treatment with water (mock) or 1 µM nlp20. Data points are indicated as dots (*n* = 6) and statistically significant differences from wild-type plants are indicated (Tukey's multiple comparisons test, ***P ≤ 0.001). Each experiment was repeated three times with similar results. Bars represent means ± s.d. of six independent experiments. Data were analyzed by Two-way ANOVA with Tukey's test. Statistically significant differences from the control are indicated (***P ≤ 0.001). Exact P values are provided in Supplementary data. All experiments were repeated at least three times with similar results.

Supplementary Table 1. Plants used in this study

Line	Species	Description	Reference
rlp23-1	*Arabidopsis*	T-DNA insertion line SALK_034225 for RLP23 in Col-0	[7]
rlp23-1/RLP23	*Arabidopsis*	*rlp23-1* T-DNA insertion line, complemented with GFP-tagged RLP23	[7]
rlp23-1/RLP23ΔIC	*Arabidopsis*	*rlp23-1* T-DNA insertion line, complemented with the GFP-tagged IC deletion version of RLP23	This study
rlp23-1/RLP23/IC$^{Cf\text{-}9}$	*Arabidopsis*	*rlp23-1* T-DNA insertion line, complemented with the GFP-tagged chimeric form of RLP23 with the Cf-9-IC domain	This study
Cf-9	*Arabidopsis*	expression line of Cf-9-GFP in Col-0 wild-type	This study
Cf-9/CT^{RLP23}	*Arabidopsis*	expression line of the GFP-tagged hybrid form of Cf-9 with RLP23 C-terminus in Col-0 wild-type	This study
Cf-9/CT^{RLP30}	*Arabidopsis*	expression line of the GFP-tagged hybrid form of Cf-9 with RLP30 C-terminus in Col-0 wild-type	This study
RE02	*N. benthamiana*	Deletion line of RE02 generated via CRISPR/Cas9	This study
RLP23	Tomato	expression line of RLP23-GFP in wild-type tomato	This study
RLP23+AtSOBIR1	Tomato	co-expression of RLP23-GFP and AtSOBIR1 in wild-type tomato	This study
RLP23/CT$^{Cf\text{-}9}$	Tomato	expression line of the GFP-tagged RLP23 chimeric receptor with Cf-9 C-terminus in wild-type tomato	This study
RLP23/CT^{EIX2}	Tomato	expression line of the GFP-tagged RLP23 chimeric receptor with EIX2 C-terminus in wild-type tomato	This study

Supplementary Table 2. Primers used for cloning

Gene	Primer name	Primer sequence (5' – 3')
RLP23	*RLP23*_F	tatggtctcatctgaacaATGTCAAAGGCGCTTTTGCA
	*RLP23*_R	ttggtctctccttACGCTTTCTGCGTTTATTCA
*RLP23*ΔIC	*RLP23*ΔIC_F	aatttactattctagtcgacctgcaggcggccgcactagtATGTCAAAGGCGCTTTTGCA
	*RLP23*ΔIC_R	ccccggtgaacagctcctcgcccttgctcaccatactagtAGCAATAACTTGTGCTATTG
RLP23-IC	*RLP23*-IC_F	CAATAGCACAAGTTATTGCTTCATACAAACCGGAGTGGCT
	RLP23-IC_R	ccccggtgaacagctcctcgcccttgctcaccatactagtACGCTTTCTGCGTTTATTCA
RLP23-CT	*RLP23*-CT_F	aaggaacacagattactgggcaatctaaatcttccttcga
	RLP23-CT_R	ccccggtgaacagctcctcgcccttgctcaccatactagtACGCTTTCTGCGTTTATTCA
*RLP23*ΔIC+TM	*RLP23*ΔIC+TM_F	aatttactattctagtcgacctgcaggcggccgcactagtATGTCAAAGGCGCTTTTGCA
	*RLP23*ΔIC+TM_R	ccccggtgaacagctcctcgcccttgctcaccatactagtTCTCCCGTTCAACACTTCTTCT
*RLP23*ΔCT	*RLP23*ΔIC_F	aatttactattctagtcgacctgcaggcggccgcactagtATGTCAAAGGCGCTTTTGCA
	*RLP23*ΔIC _R	ccccggtgaacagctcctcgcccttgctcaccatactagtCCAGTAATCTGTGTTCCTTGT
RLP23^{2mut}	RLP23_S872A_Y873F_F	ACAAGTTATTGCTgCAtCAAACCGGAGTGGC
	RLP23_S872A_Y873F_R	GCCACTCCGGTTTGaATGcAGCAATAACTTGT
*RLP30*ΔIC	*RLP30*ΔIC_F	aatttactattctagtcgacctgcaggcggccgcactagtATGATTCCAAGCCAATCTAA
	*RLP30*ΔIC_R	ccccggtgaacagctcctcgcccttgctcaccatactagtGAAGATATGTCCAATAACTAA
RLP30-IC	*RLP30*-IC_F	AGTTATTGGACATATCTTCTTTACTGCACACAAACACGAG
	RLP30-IC_R	ccccggtgaacagctcctcgcccttgctcaccatactagtACGAGCACTTGTGGTGACTA
RLP30-CT	*RLP30*-CT_F	CCCGGAATCAGCTATCAGGTCACATCCCTCGAGATCTTGG
	RLP30-CT_R	ccccggtgaacagctcctcgcccttgctcaccatactagtGAAGATATGTCCAATAACTAA
*RLP30*ΔIC+TM	*RLP30*ΔIC+TM_F	aatttactattctagtcgacctgcaggcggccgcactagtATGATTCCAAGCCAATCTAA
	*RLP30*ΔIC+TM_R	ccccggtgaacagctcctcgcccttgctcaccatactagtGTCTGAGGTTGTCCATGAATG
*RLP30*ΔCT	*RLP30*ΔIC_F	aatttactattctagtcgacctgcaggcggccgcactagtATGATTCCAAGCCAATCTAA
	*RLP30*ΔIC _R	ccccggtgaacagctcctcgcccttgctcaccatactagtGACCTGATAGCTGATTCCGGGA
RLP30^{4mut}	*RLP30*_F	ATGATTCCAAGCCAATCTAATTCC
	RLP30-T761A_F	TATCTTCTTTGCTGCACACAAACACGAG
	RLP30-T761A_R	CTCGTGTTTGTGTGCAGCAAAGAAGATA
	RLP30-Rmut-all	ACGAGCAGCTGCGGCGACTACTCT
Cf-9	*Cf-9*_F	tatggtctcatctgaacaATGGATTGTGTAAAACTTGTA
	*Cf-9*_R	ttggtctctccttATATCTTTTCTTGTGCTTTTTC
Cf-9-IC	*Cf-9*-IC_F	CAATAGCACAAGTTATTGCTCAACTCAATATCCAGCATGG
	Cf-9-IC_R	ccccggtgaacagctcctcgcccttgctcaccatactagtATATCTTTTCTTGTGCTTTTTC
Cf-9-CT	*Cf-9*-CT_F	TTGTTGGATGCATCCCCAAAGGAAAACAATTTGATTCGTTC

	Cf-9-CT_R	ccccggtgaacagctcctcgcccttgctcaccatactagtATATCTTTTCTTGTGCTTTTTC
EIX2	*EIX2*_F	tatggtctcatctgaacaATGGGCAAAAGAACTAATCCA
	*EIX2*_R	ttggtctctccttGTTCCTTAGCTTTCCCTTCAGT
EIX2-IC	*EIX2*-IC_F	CAATAGCACAAGTTATTGCTGTCAACCGTTCGTGGAGGAAT
	EIX2-IC_R	ccccggtgaacagctcctcgcccttgctcaccatactagtGTTCCTTAGCTTTCCCTTCAGT
EIX2-CT	*EIX2*-CT_F	GAATTCCATCAAGCACTCAACTCAGAGTTTCGATAGATCATCC
	EIX2-CT_R	ccccggtgaacagctcctcgcccttgctcaccatactagtGTTCCTTAGCTTTCCCTTCAGT
OsRLP1-IC	*OsRLP1*-IC_F	CAATAGCACAAGTTATTGCTGA
	OsRLP1-IC _R	ccccggtgaacagctcctcgcccttgctcaccatactagtGTTACCTTCCGTAGTATATCCA
OsRLP1-CT	*OsRLP1*-IC_F	GCATATGGATTATCTGTCGTTTCTGCCATCCTCATATTCAG
	OsRLP1-IC_R	ccccggtgaacagctcctcgcccttgctcaccatactagtGTTACCTTCCGTAGTATATCCA
PaRLP1-IC	*PaRLP1*_F	CAATAGCACAAGTTATTGCTCC
	*PaRLP1*_R	ccccggtgaacagctcctcgcccttgctcaccatactagtCCTTCTGAATCTGGACAACTTG
PaRLP1-CT	*PaRLP1*-IC_F	ATATCTTTTTTGCTTGCCTTGAATCTATCAGGA
	PaRLP1-IC_R	ccccggtgaacagctcctcgcccttgctcaccatactagtCCTTCTGAATCTGGACAACTTG
MoNLP1	*MoNLP1*_F	CGGAATTCCTCGCGGCCCGGGACGTCATC
	*MoNLP1*_R	GCTCTAGACCGAAAACCTTGGCCAGGTTGTTCTG

Supplementary Table 3. Primers used for RT-PCR

Gene	Primer name	Primer sequence (5' – 3')
SlEF1α	*SlEF1α*_F	CGTGAGCGTGGTATCACCATT
	*SlEF1α*_R	GGTAGACCTCTCAATCATGTT
SlPR1a	*SlPR1*_F	TGGTATTAGCCATATTTCAC
	*SlPR1*_R	CCAGTTGCCTACAGGATC
SlEDS1	*SlEDS1*-F	GGAATTGAAGTCAGAGATGAGCTAA
	SlEDS1-R	AAAGTTCCAGCAAAAGCAAAAA
SlNRC2	*SlNRC2*-F	GACGTGGTTGGATTTGACGAAG
	SlNRC2-R	GTTACAATTCTCAGGGTGGTCTC
SlNRC4a	*SlNRC4a* -F	GCTAATCAATTTTATGCTTCCACTG
	SlNRC4a -R	CGTGATCAATGACATCGTCCTC
SlRIPK	*SlRIPK*_F	GTCATTTGACTGCAGCTAGTGA
	*SlRIPK*_R	GGATCTCTTGAAACCATTTTGC
PaCML42	*PaCML42*_F	CATCAATCATTGGACAGCAGTT
	*PaWRKY22*_R	ACCTTGAATGCCTCTGACAAAT
PaWRKY22	*PaCML42*_F	GTCTAAAGGGATGTTTGGCAAG
	*PaWRKY22*_R	TTTAGGCATAGATGGCTTGGTT
PaRAP2.6	*PaRAP2.6*_F	ACTGTGCTCATGGTGATTTGTC
	*PaRAP2.6*_R	GCTCTTCTCTGCAGGTTTCATT
PaPR1	*PaPR1*_F	TCAATGCCCACAATAATGCTCG
	*PaPR1*_R	TAAGATCACCACTACCTCCTGC
PaEF1α	*PaEF1α*_F	CCTGGACATCGTGACTTTATCA
	*PaEF1α*_R	GTCCATCTTGTTACAGCAGCAG
OsWRKY70	*OsWRKY70*_F	CCGCTGCTGTTTTGATCATCT
	*OsWRKY70*_R	CCGCTGCTGTTTTGATCATCT
OsPAL	*OsPAL*_F	CTACCCGCTGATGAAGAAGC
	*OsPAL*_R	GAACCTTGTTCAGCTCCTCG
OsPR1a	*OsPR1a*_F	TCGTATGCTATGCTACGTGTTT
	*OsPR1a*_R	CACTAAGCAAATACGGCTGACA
OsMAPK6	*OsMAPK6*_F	CGCACGCTCAGGGAGATC
	*OsMAPK6*_R	GGTATGATATCCCTTATGGCAACAA
OsActin	*OsActin*_F	TTATGGTTGGGATGGGACA
	*OsActin*_R	AGCACGGCTTGAATAGCG

Supplementary Table 4. Accession numbers of genes used in this study

**Gene**	**ID number**
Cf-9	*Solyc01g096480*
EIX2	*Solyc07g008630.1.1*
MaNLP1	OM964796.1
MoNLP1	XP_003715954
OsActin	*Os12g0547500*
OsMAPK6	*Os06g0154500*
OsPAL	*Os02g0626400*
OsPR1a	*Os03g0820600*
OsRLP1	*LOC_Os06g04830*
OsWRKY70	*Os05g0474800*
PaCML42	*XP_011018929.1*
PaEF1α	*KAH8505600.1*
PaPR1	*XP_002308281.1*
PaRAP2.6	*KAH8506047.1*
PaRLP1	*Potri.005G012100*
PaWRKY22	*XP_061961387.1*
RLP23	*AT2G32680.1*
RLP30	*AT3G05360.1*
SlEDS1	*Solyc02g032850.3*
SlEF1α	*Solyc06g005060*
SlNRC2	*Solyc10g047320.2*
SlNRC4a	*Solyc04g007070.2.1*
SlPR1a	*Solyc01g106610*
SlRIPK	*Solyc06g062920.3*

Figure S1b

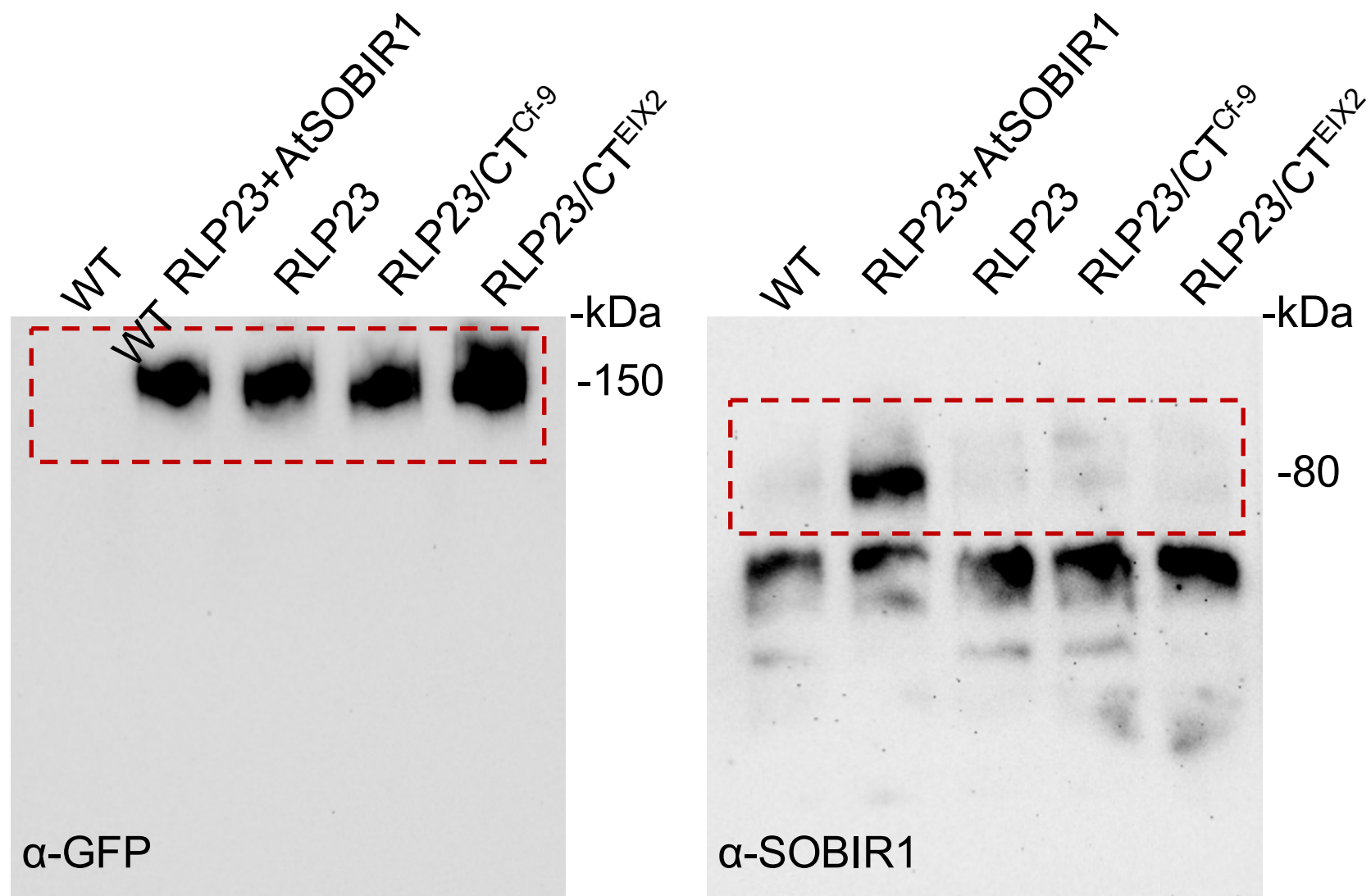
WT
RLP23+AtSOBIR1
RLP23
RLP23/CTCf-9
RLP23/CTEIX2
-kDa
-150
α-GFP
WT
RLP23+AtSOBIR1
RLP23
RLP23/CTCf-9
RLP23/CTEIX2
-kDa
-80
α-SOBIR1

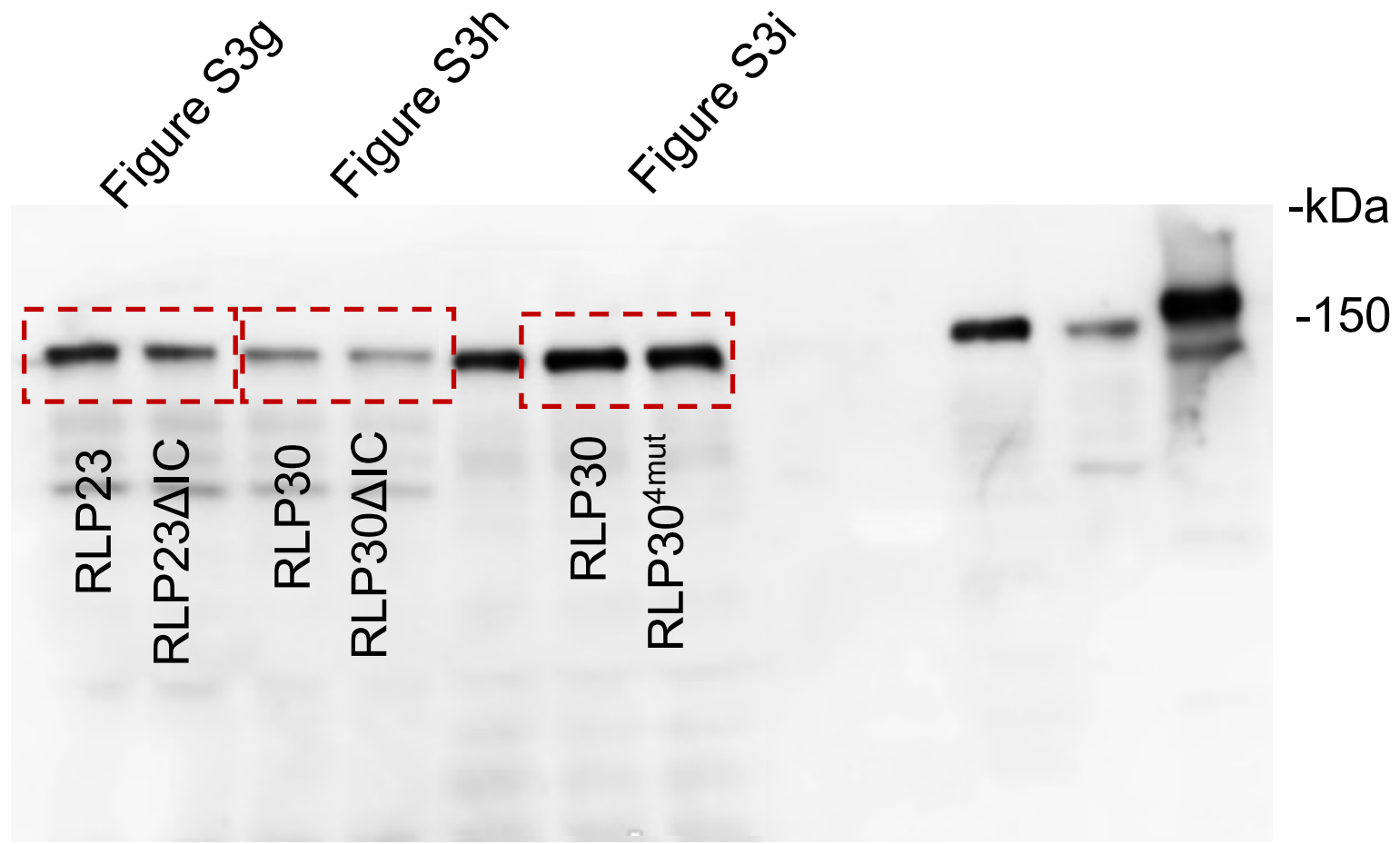
Figure S3g
Figure S3h
Figure S3i
-kDa
-150
RLP23
RLP23ΔIC
RLP30
RLP30ΔIC
RLP30
RLP304mut

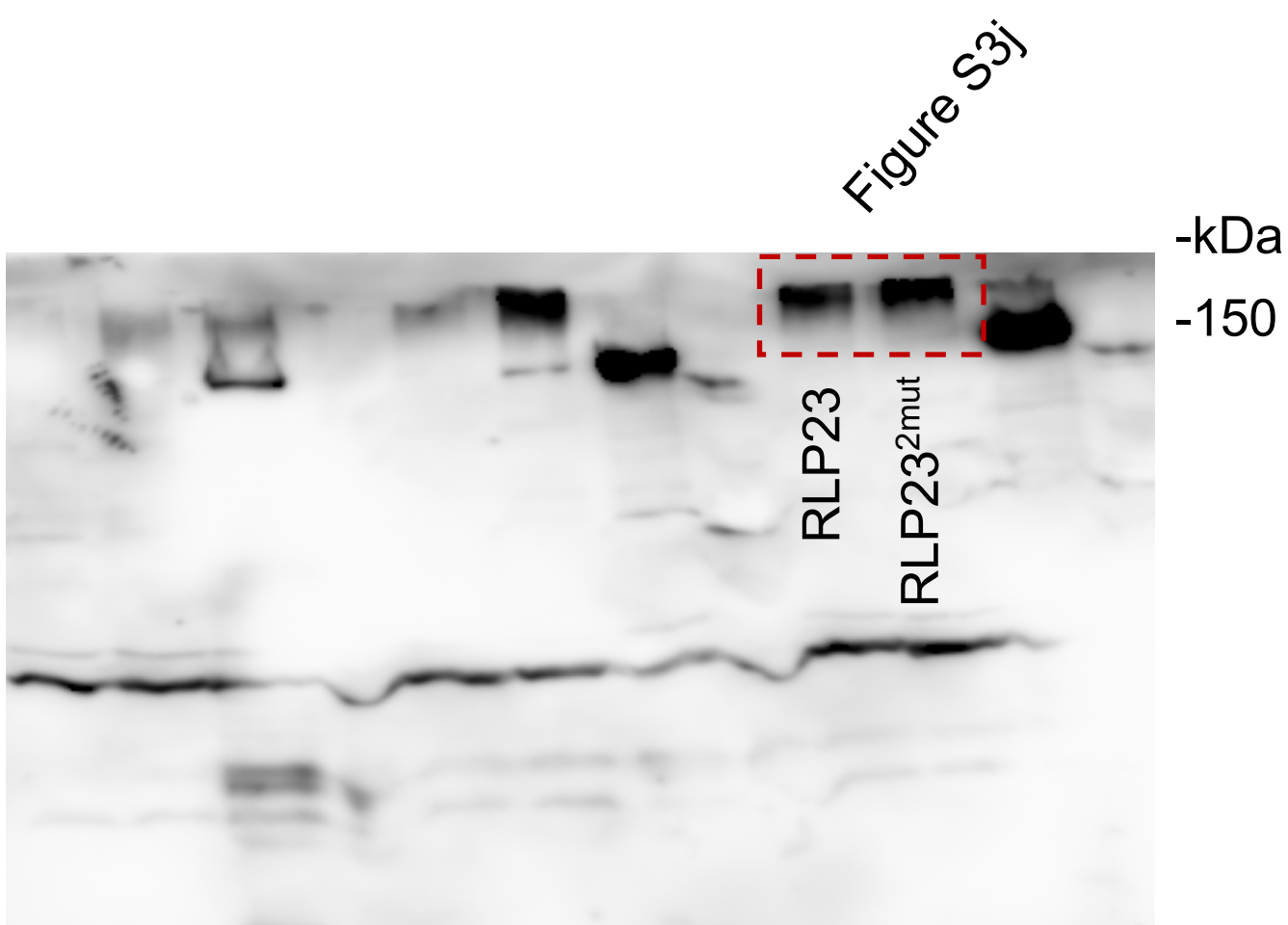
Figure S3j
-kDa
-150
RLP23
RLP232mut
