# Supplementary material for: Engineered pattern recognition receptors enhance broad-spectrum plant resistance
Source: Nat Biotechnol. 2025 Oct 13;44(10):1715–21. doi: 10.1038/s41587-025-02858-8 (PMC13654806; doi:10.1038/s41587-025-02858-8)

Figure 3a

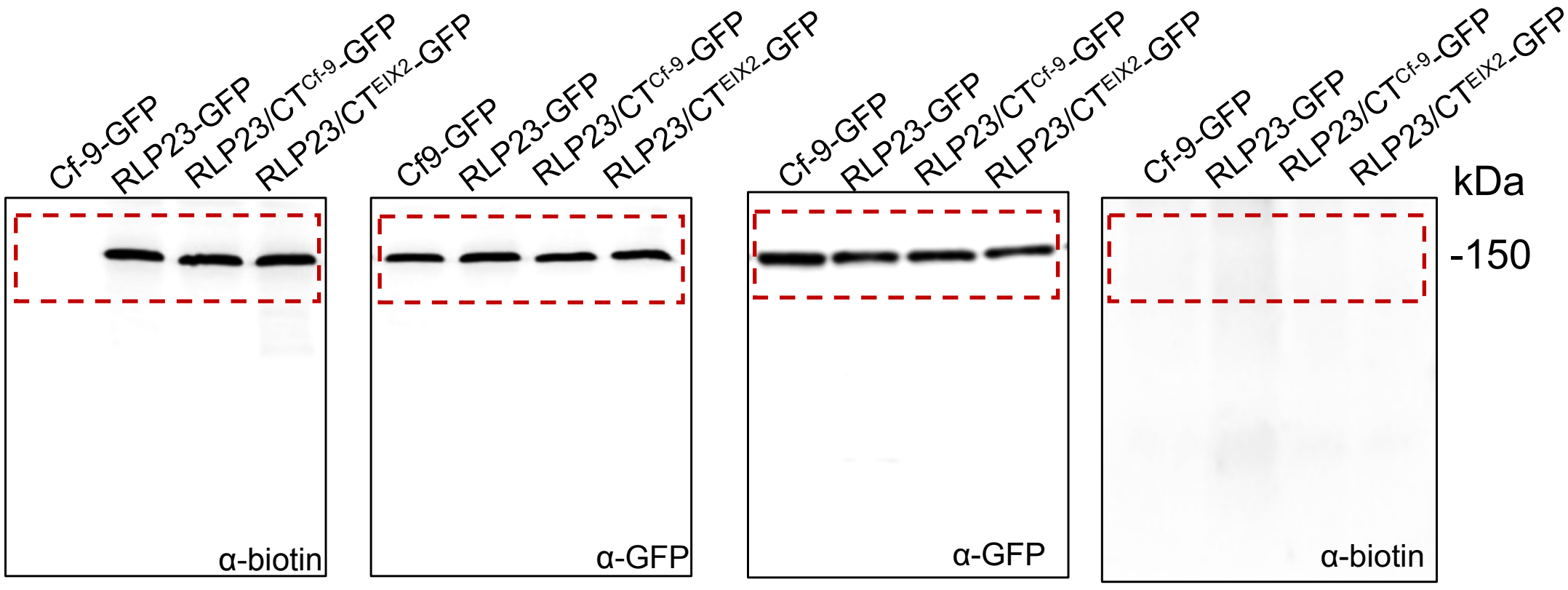

Figure 3b

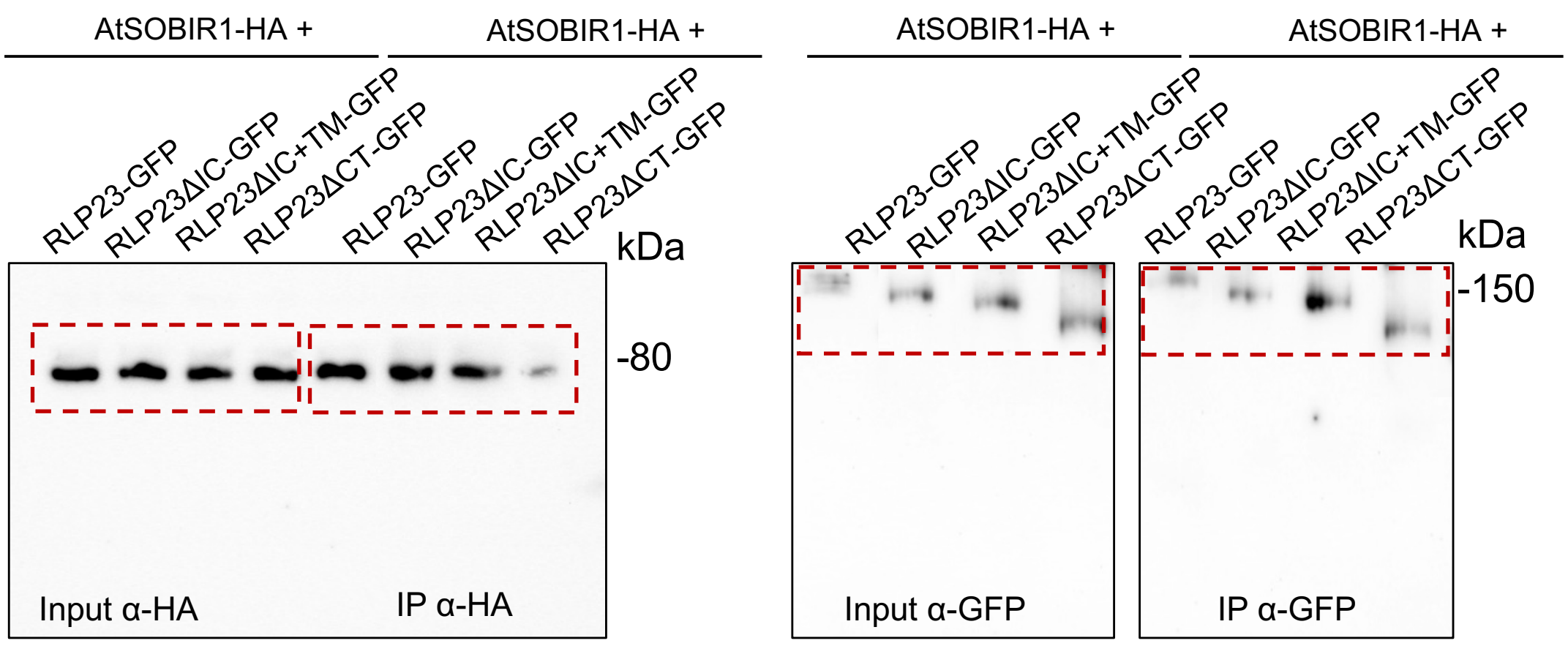

Figure 3c

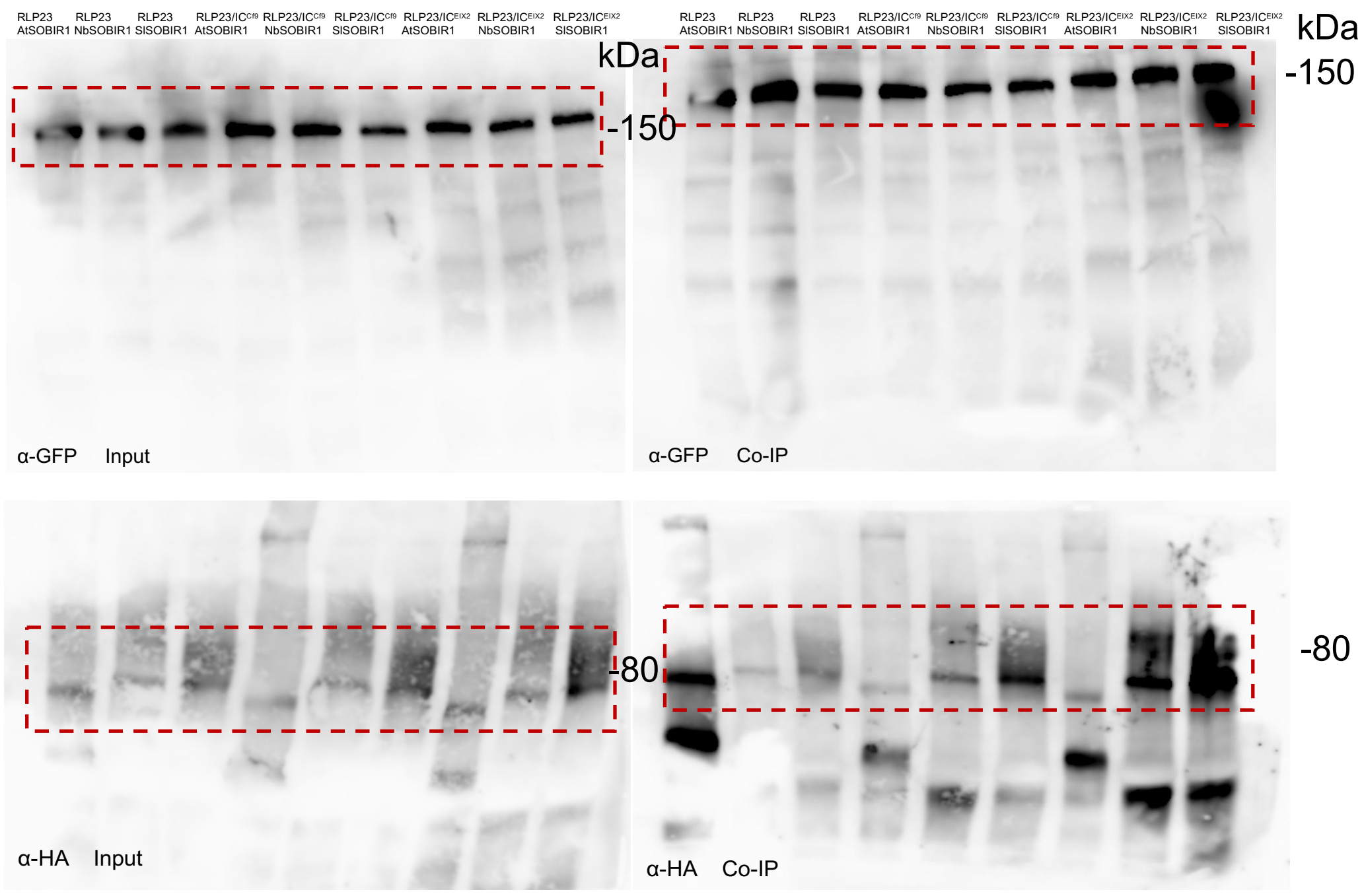

Supplement: Supplementary file 3 [file 41587_2025_2858_MOESM5_ESM.pdf]
